# Fibroelastic Remodelling of the Endocardium on the Right Side of the Heart: Endothelial-to-Mesenchymal Transition in Pulmonary Atresia With Intact Ventricular Septum

**DOI:** 10.1093/ejcts/ezag143

**Published:** 2026-04-03

**Authors:** Julia Gaal, Daniel Diaz-Gil, Alexa von Mueffling, Cindy Zajac, Viktoria Weixler, Umji Lee, Andrei-Antonio Caracioni, Gregor Gierlinger, Joachim Photiadis, John E Mayer, Sitaram M Emani, Pedro J del Nido, Ingeborg Friehs

**Affiliations:** Department of Cardiac Surgery, Boston Children’s Hospital, Boston, MA 02115, United States; Department of Congenital Heart Surgery-Pediatric Heart Surgery, German Heart Center, Charité - Universitätsmedizin Berlin, 13353 Berlin, Germany; Department of Cardiology, Boston Children’s Hospital, Boston, MA 02115, United States; Department of Pediatrics, Harvard Medical School, Boston, MA 02115, United States; Department of Cardiac Surgery, Boston Children’s Hospital, Boston, MA 02115, United States; Department of Cardiac Surgery, Boston Children’s Hospital, Boston, MA 02115, United States; Department of Prenatal Medicine, University Hospital of Giessen and Marburg, Justus Liebig University Giessen, 35385 Giessen, Germany; Department of Congenital Heart Surgery-Pediatric Heart Surgery, German Heart Center, Charité - Universitätsmedizin Berlin, 13353 Berlin, Germany; Department of Cardiac Surgery, Boston Children’s Hospital, Boston, MA 02115, United States; Department of Surgery, Harvard Medical School, Boston, MA 02115, United States; Department of Cardiac Surgery, Boston Children’s Hospital, Boston, MA 02115, United States; Division of Cardiac Surgery, Medical University of Graz, 8036 Graz, Austria; Division of Pediatric and Congenital Heart Surgery, Kepler University Hospital, 4021 Linz, Austria; Department of Congenital Heart Surgery-Pediatric Heart Surgery, German Heart Center, Charité - Universitätsmedizin Berlin, 13353 Berlin, Germany; Department of Cardiac Surgery, Boston Children’s Hospital, Boston, MA 02115, United States; Department of Surgery, Harvard Medical School, Boston, MA 02115, United States; Department of Cardiac Surgery, Boston Children’s Hospital, Boston, MA 02115, United States; Department of Surgery, Harvard Medical School, Boston, MA 02115, United States; Department of Cardiac Surgery, Boston Children’s Hospital, Boston, MA 02115, United States; Department of Surgery, Harvard Medical School, Boston, MA 02115, United States; Department of Cardiac Surgery, Boston Children’s Hospital, Boston, MA 02115, United States; Department of Surgery, Harvard Medical School, Boston, MA 02115, United States

**Keywords:** pulmonary atresia with intact ventricular septum, endocardial fibroelastosis, fibroelastic remodelling, fibrogenic activation, endothelial-to-mesechnymal transition, flow disturbance

## Abstract

**Objectives:**

Patients with pulmonary atresia/critical pulmonary stenosis with intact ventricular septum (PA/cPS-IVS) show fibrous subendocardial tissue of unclear origin accompanied by right ventricular (RV) hypoplasia. While in hypoplastic left heart syndrome, it is known that flow-induced endothelial-to-mesenchymal transition (EndMT) of endocardial endothelial cells (EECs) is the source of this fibrous tissue, it remains unclear whether similar mechanisms exist in PA/cPS-IVS.

**Methods:**

We analysed 13 PA/cPS-IVS patients who underwent staged ventricular rehabilitation surgery aimed at preserving RV function between March 2021 and July 2025 at Boston Children’s Hospital. Resected tissue was examined for the degree of fibrosis and elastin and the presence of active EndMT by histology, immunohistochemistry, and flow cytometry. To mimic human disease conditions, isolated EECs were exposed to pathological flow and compared with physiological flow conditions.

**Results:**

Flow disturbances across the pulmonary and/or tricuspid valves were present in all patients. Resected RV tissue revealed an active subendocardial fibroelastic remodelling process, infiltrating into the underlying myocardium. Patients showed RV diastolic dysfunction, as evidenced by elevated filling pressures, suggesting a pathophysiological role of fibroelastic remodelling of the endocardium. Mimicking the human disease, exposure of isolated EECs to pathological flow conditions induced loss of endothelial characteristics and transition towards a mesenchymal phenotype through EndMT.

**Conclusions:**

In PA/cPS-IVS patients, restrictive RV physiology and diastolic dysfunction are likely driven by infiltrative fibroelastic remodelling caused by localized fibrogenic activation of EECs through EndMT in response to flow disturbances from valvular defects. Fibrogenic pathway activation may represent a promising therapeutic target in PA/cPS-IVS.

## Graphical abstract

**Figure:**
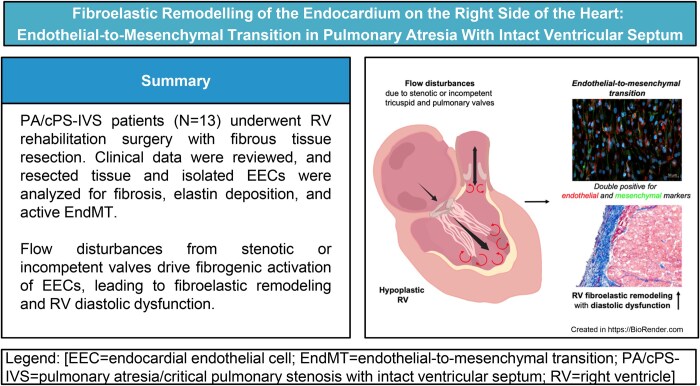

## Introduction

Pulmonary atresia/critical pulmonary stenosis with intact ventricular septum (PA/cPS-IVS) is a rare cyanotic congenital heart defect characterized by a complete or near-complete obstruction of the right ventricular outflow tract (RVOT). Patients with PA/cPS-IVS typically present with varying degrees of tricuspid valve (TV) and right ventricular (RV) hypoplasia, along with subendocardial fibrous tissue accumulation of unknown origin within the small RV.^1–9^ At Boston Children’s Hospital, for patients with adequate RV size, we aim to recruit the small RV, which involves resection of RV muscle bundles and fibrous tissue, RVOT reconstruction, and TV repair. This approach promotes RV growth, increases RV volume, and improves TV mobility, thereby aiming to create a more physiological flow profile with 2-ventricle (2 V) or one-and-a-half-ventricle (1.5 V) circulation.^10^^,^^11^

The left-sided haemodynamic equivalent disease, hypoplastic left heart syndrome, is often associated with fibroelastic subendocardial thickening, known as endocardial fibroelastosis (EFE).^12^^,^^13^ The presence of EFE restricts ventricular growth and function, limiting the LV’s use as a systemic ventricle.^12^^,^^14–16^ We have previously demonstrated that EFE is driven by endothelial-to-mesenchymal transition (EndMT), in which endothelial cells undergo fibrogenic activation, losing their endothelial characteristics and acquiring mesenchymal markers.^17^^,^^18^ This mechanism is transforming growth factor-beta (TGF-β) mediated through the transcription factor Slug/Snail.^13^ EndMT is a hallmark of cellular plasticity and plays a physiological role in the development of heart valves.^19^ However, we have established that flow disturbances from defective valves and mechanical stretch from ventricular pressure overload trigger this process pathologically.^17^^,^^18^^,^^20–25^ We aimed to determine whether EndMT is the underlying mechanism driving subendocardial fibrous tissue remodelling in PA/cPS-IVS and to assess whether pathological intracardiac flow disturbances mediate this process.

## Methods

A detailed methods section is provided in **File S1**.

### Ethics statement

This study was approved by the institutional review board of Boston Children’s Hospital (P0038762 and P00026224, approved 29 April 2021) and conducted in accordance with its ethical standards. Due to the retrospective nature of this study, the patient consent was waived.

### Patients

Patients with PA/cPS-IVS who underwent staged RV rehabilitation surgery involving fibrous tissue resection at Boston Children’s Hospital between March 2021 and July 2025 were included. Clinical data, procedural details, and short-term follow-up were collected from medical records. Cardiovascular-healthy post-mortem RV tissue (*N* = 3) served as controls.

### Histologic assessment

Tissue samples were stained with hematoxylin and eosin (H&E) for overall morphology, Masson’s trichrome (MT) for the degree of collagen, and Elastica van Gieson (EVG) for elastin deposition. The percentage area of fibrosis and elastin deposition was calculated by dividing the sum of fibrotic or elastic areas by the total tissue area. Immunohistochemistry (IHC) assessed EndMT by the co-expression of vascular endothelial cadherin (VE-cadherin), an endothelial marker, and vimentin, a mesenchymal marker. Nuclear co-localization of VE-cadherin-positive cells with phosphorylated suppressor of mothers against decapentaplegic 2/3 (pSMAD2/3) or Slug/Snail suggested active TGF-β-mediated EndMT.

### Cell culture and endothelial cell function assay

Healthy EECs (HEECs) from cardiovascular-healthy paediatric post-mortem donors (*N* = 3) and PA/cPS-IVS-derived endocardial endothelial cells (PA/cPS-IVS-EECs) were isolated and cultured. After 48 hours under pathological static conditions, endothelial cell function was determined using acetylated low-density lipoprotein labelled with 1,1′-dioctadecyl-3,3,3′,3′-tetramethylindocarbocyanine perchlorate (DiI-Ac-LDL) uptake.

### *In vitro* flow experiments

PA/cPS-IVS-EECs were seeded in ibidi slides (ibidi USA, Inc., Fitchburg, WI) and exposed to physiological (7.5 dynes/cm^2^) and pathological (0 and 40 dynes/cm^2^) flow for 48 hours.^17^ The co-expression of endothelial cluster of differentiation 31 (CD31) and mesenchymal vimentin indicated EndMT.

### Flow cytometry

Surface markers CD31 (endothelial) and fibroblast-surface marker (mesenchymal), along with intracellular nuclear factor of activated T cells 1 (NFATC1) (endocardial specific) and vimentin (mesenchymal), were analysed by flow cytometry to determine EndMT in isolated single cells. To assess nonspecific binding, isotype controls were used, respectively.

### Real-time qRT-PCR

Real-time qRT-PCR was performed with SYBR Green Master Mix on a QuantStudio 3 Real-Time PCR System. The expression of target genes (Ct values) was normalized to glyceraldehyde-3-phosphate dehydrogenase (ΔCt) and then expressed as relative expression = 1000 × 2^ (−ΔCt). All samples were analysed in technical triplicate.

### Statistical analysis

Statistical analyses were performed with GraphPad Prism 10. Continuous patient-derived variables are presented as median (IQR, 25th-75th percentile), continuous cell culture data as mean with SD, and categorical variables as frequency (%). All patient-derived data were summarized using descriptive statistics. All cell data derived from human samples were analysed for comparisons using parametric tests after confirming normal distribution: an unpaired *t*-test for 2-group comparisons or 1-way ANOVA with Bonferroni correction for multiple groups, as appropriate. Cell culture experiments were performed in biological triplicate, and *P* < .05 was considered significant.

## Results

### Patients

During the study period, a total of 28 patients, with adequate RV size, underwent surgery for 1.5 or 2 V circulation at our institution. A total of 17 patients underwent additional fibrous tissue resection, whereof we obtained tissue from 13 consecutive patients (13/17) for detailed pathomechanistic analysis. Resected tissue from 4 patients was not made available to us but was described as fibrous tissue through routine pathological analysis. The median age at surgery was 3.5 years (IQR: 1.0-5.9), with a median follow-up time of 12.4 months (IQR: 1.9-33.5). At the time of fibrous tissue resection, 69.2% (9/13) were undergoing initial rehabilitation surgery via ventricular recruitment. The remaining 4 patients have already had recruited RVs (1.5 V). Preoperatively, 61.5% (8/13) had at least moderately hypoplastic RVs. Furthermore, patients showed a median right ventricular end-diastolic pressure (RVEDP) of 12 mmHg (IQR: 11-14), suggesting reduced RV diastolic compliance. However, mean pulmonary artery pressures (mPAP) and pulmonary vascular resistance index (PVRI) were normal with a median of 13.5 mm Hg (IQR: 13.0-16.3) and a median of 2.0 iWu (IQR: 1.7-2.6). Our cohort exhibited RV pressures at systemic levels with an RV systolic pressure to systemic pressure ratio of 1.0 (IQR: 0.4-1.4) (**Table S1**).

**Figure 1.**
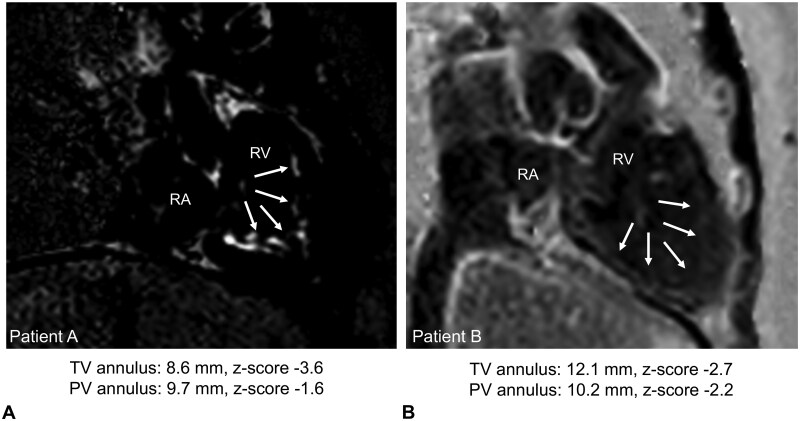
**Representative preoperative MRI studies from patients with PA/cPS-IVS.** Preoperative MRIs from 2 patients with PA/cPS-IVS demonstrated prominent, confluent subendocardial late gadolinium enhancement throughout the RV cavity (arrows), with an infiltrative growth pattern extending into the underlying myocardium. Corresponding echocardiographic valve measurements are shown below. Abbreviations: PA/cPS-IVS, pulmonary atresia/congenital pulmonary stenosis with intact ventricular septum; RA, right atrium; RV, right ventricle.

The available preoperative MRIs (5/13) showed a confluent subendocardial late gadolinium enhancement (LGE) layer in the RV cavity, TV leaflets, and papillary muscles. In 60.0% (3/5) of those patients, LGE also demonstrated infiltration into the underlying myocardium (**Figure 1**). In patients where preoperative imaging did not indicate endocardial changes, diagnosis was performed intraoperatively at the cardiac surgeon’s discretion.

On preoperative echocardiography, all patients had flow disturbances (**Table 1**). At the valvular level, 92.3% (12/13) had a hypoplastic TV, with a median annulus diameter z-score of −2.8 (IQR: −4.0 to −2.6). A severely stenotic to nearly atretic TV was observed in 30.8% (4/13), with regurgitation in 71.4% (10/14). Among the patients who underwent pulmonary valve (PV) perforation and balloon dilation before surgery (7/14), the median PV annulus z-score was −1.9 (IQR: −4.4 to −0.9), and 85.7% (6/7) developed at least moderate pulmonary regurgitation. Detailed information is provided in the **Table S1**.

**Table 1.** Detailed preoperative information of included patients with PA/cPS-IVS

Patient	Age (years)	Diagnosis	Previous procedures	Symptoms/indication for surgery	Localization of fibrous tissue
01	0.96	PA-IVS with subvalvar muscular narrowing, PR (minimal antegrade flow), TR (to-and-fro flow), cavity flow acceleration	2× PV BD, PDA stent, BD stent, RVOT reconstruction, PDA ligation, mBTTS	Progressive cyanosis, tachypnea	RA, RV parietal wall, anterior papillary muscles, conal septum, apex
02	4.34	Near membranous PA-IVS, PR, TR (jet)	PV BD, PDA stent, ASD stent, BD stent, RVOT reconstruction, RV muscle bundle resection, PFO restriction, BDG	Low cardiac output, poor exercise tolerance, cyanosis	RV free wall and septum, apex, papillary muscles
03	5.11	Membranous PA-IVS, PA, severe TS to nearly atretic (no significant antegrade flow)	mBTTS, BDG	Cyanosis, poor exercise tolerance	RV, papillary muscles
04	9.90	Membranous PA-IVS, PA, TS/TR (no significant inflow)	BDG, Fontan	Failing Fontan with protein losing enteropathy and growth faltering	RV free wall, basal and mid septum, RV muscle bundles
05	3.45	Membranous PA-IVS, PA, TR (miniscule/absent antegrade flow, jet)	mBTTS, BDG, PDA ligation, BTTS takedown	Growth faltering, cyanosis, poor exercise tolerance	RV base and inferior wall, TV leaflets
06	4.39	Membranous PA-IVS, PA, TR (to-and-fro flow)	BDG, atrial septostomy, PDA ligation, 2× PDA stent, BD PDA stent	Cyanosis	RV
07	0.96	Critical PS-IVS (near atretic), PR (jet, flow acceleration below PV), TR	PV BD, PDA stent	Elective RV recruitment	RVOT
08	0.61	Membranous PA-IVS, PR (miniscule/absent antegrade flow, diastolic pulmonary flow via paravalvar leak), TS/TR (miniscule/absent antegrade flow)	PV BD, 2× PDA stent, BD LPA, BAS	Cyanosis	RV, RA, orifice and 3 leaflets of TV
09	6.74	Membranous PA-IVS, PA (miniscule/absent inflow and outflow), functional properties of TV were not possible	BDG, atrial septostomy, PDA stent, PDA stent ligation	Cyanosis, poor exercise tolerance	RV inferior and anterior wall, septum, transmural apical anterior wall
10	1.02	Membranous PA-IVS with infindibular muscular narrowing, PR (to-and-fro flow), TS/TR (minimal antegrade flow), cavity flow acceleration	PV BD, PDA stent, 2x PDA stent BD, BAS	Growth faltering, cyanosis	RV, RVOT
11	2.49	Membranous PA-IVS, PR (to-and-fro flow), TS	BAS, 2× PDA stent, ASD stent, BDG, LPA plasty, PDA ligation, RVOT reconstruction, RV fibrous tissue and muscle bundle resection, TV repair, fenestrated closure ASD	Cyanosis	Entire RV cavity, transmural midventricular anterior and posterior septum
12	0.38	Membranous PA-IVS, PR (jet, to-and-fro flow), TS/TR (jet)	BD PDA, BAS, 2× PV BD	Progressive cyanosis	RV
13	5.13	Membranous PA-IVS, PR, TR (2× jets)	2× PV BD, 2× PDA stent, RVOT stent, BAS, PDA/RVOT stent removal, RVOT reconstruction, RV muscle bundle resection, TV repair, BDG, ASD closure	Progressive cyanosis	RV free wall, apex, TV leaflets, and papillary muscles

Abbreviations: ASD, atrial septal defect; BAS, balloon atrial septostomy; BD, balloon dilation; BDG, bidirectional Glenn; LPA, left pulmonary artery; MPA, main pulmonary artery; mBTTS, modified Blalock-Taussig-Thomas shunt; PA-IVS, pulmonary atresia with intact ventricular septum; PDA, patent ductus arteriosus; PFO, patent foramen ovale; PR, pulmonary regurgitation; PS, pulmonary stenosis; PS-IVS, pulmonary stenosis with intact ventricular septum; PV, pulmonary valve; RA, right atrium; RPA, right pulmonary artery; RV, right ventricle; RVOT, right ventricular outflow tract; TR, tricuspid regurgitation; TS, tricuspid stenosis; TV, tricuspid valve.

A total of 38.5% (5/13) of patients experienced recurrence of fibrous tissue after a median of 27.3 months (IQR: 10.3-48.7), associated with haemodynamically significant flow disturbances due to stenotic or incompetent TV in 80.0% (4/5) of patients, as evidenced by a tricuspid inflow jet detected on echocardiography at last follow-up. Detailed follow-up information is available in the **Table S2**.

### Subendocardial fibrotic tissue accumulation identified as endocardial fibroelastosis

**Figure 2.**
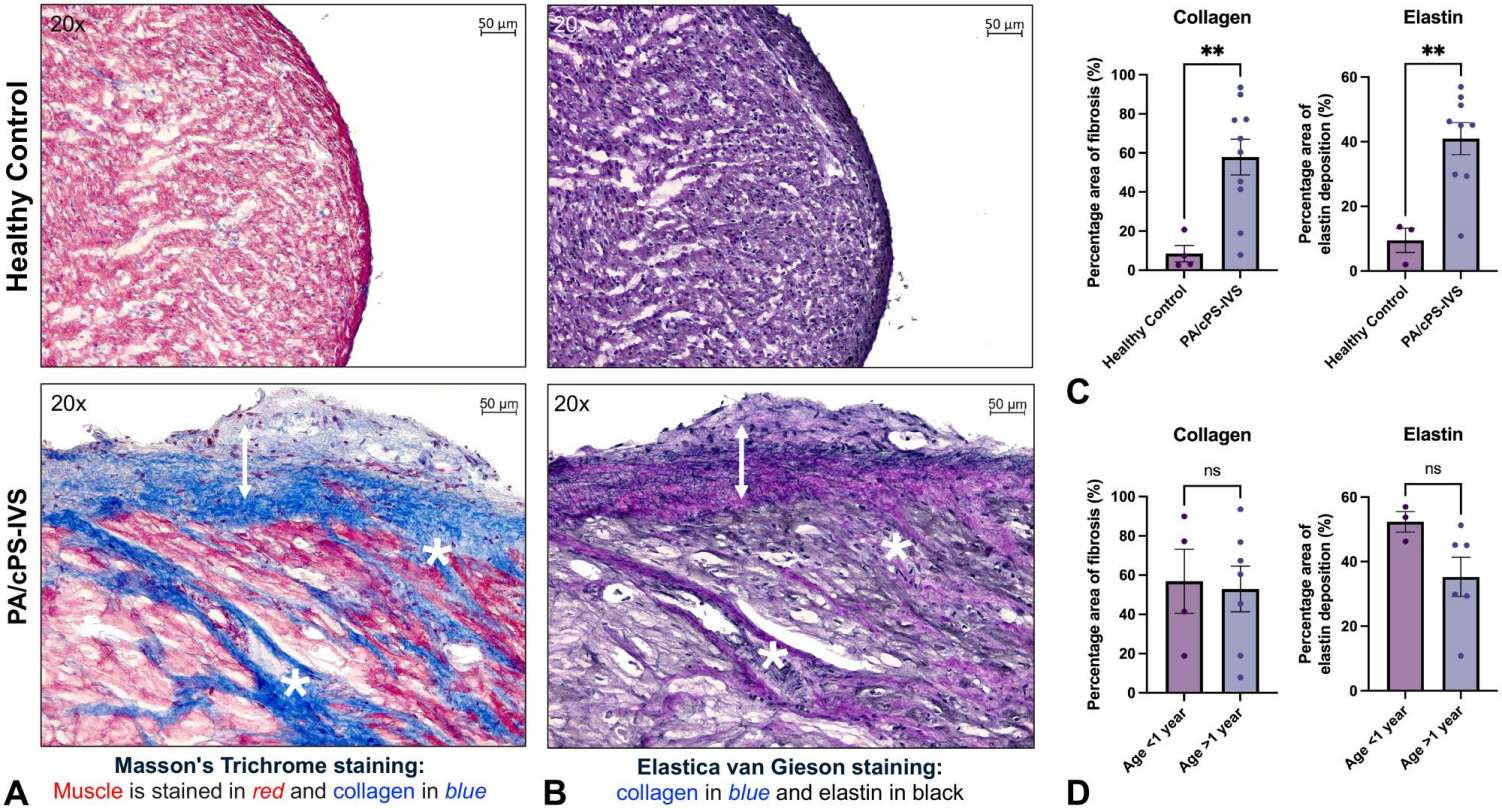
**Tissue samples from PA/cPS-IVS patients were stained to assess fibrosis and elastin content.** (A) PA/cPS-IVS samples showed a thick layer of subendocardial tissue proliferation (white arrow) with infiltrative growth patterns of collagen (*) into the underlying myocardium (*). (B) Corresponding areas identified elastin deposition with the same growth patterns. The disproportionate tissue proliferation predominantly consists of an avascular, hypocellular matrix with excessive, organized layers of elastin and collagen. Healthy control remains negative for fibroelastic remodelling. (C) Quantitative analysis showed significantly greater percentages of fibrosis and elastin in PA/cPS-IVS tissue compared to healthy controls. (D) There was no significant difference in tissue composition between age groups (<1 vs >1 year). Unpaired *t*-test analysis. Graphs represent the mean with SE. Scatter dot plots are biological replicates (N). Ns *P* > .05, **P < .01. Abbreviations: PA/cPS-IVS, pulmonary atresia/congenital pulmonary stenosis with intact ventricular septum.

In all 13 patients, histological analysis revealed disproportionate accumulation of subendocardial tissue. H&E staining showed avascular but cellular subendocardial tissue (**Figure S1**), with thick, organized layers of collagen and elastin fibres on MT and EVG stains infiltrating into the underlying myocardium (**Figure 2A and B**). The composition and growth pattern were characteristic of fibroelastic remodelling, previously described in the setting of EFE.^17^^,^^20^^,^^21^^,^^23^^,^^25^ In contrast, healthy RV tissue samples displayed a thin endocardial layer clearly separated from the underlying myocardium and remained negative for fibroelastic remodelling (**Figure 2A and B**). Quantitative analysis of tissue sections demonstrated significantly more fibrosis (*P* = .007) and elastin areas (*P* = .006) relative to the total tissue area in PA/cPS-IVS compared to controls (**Figure 2C**). Fibroelastic remodelling did not vary significantly with age (fibrosis: *P* = .846; elastin: *P* = .104; **Figure 2D**).

### Presence of active EndMT

**Figure 3.**
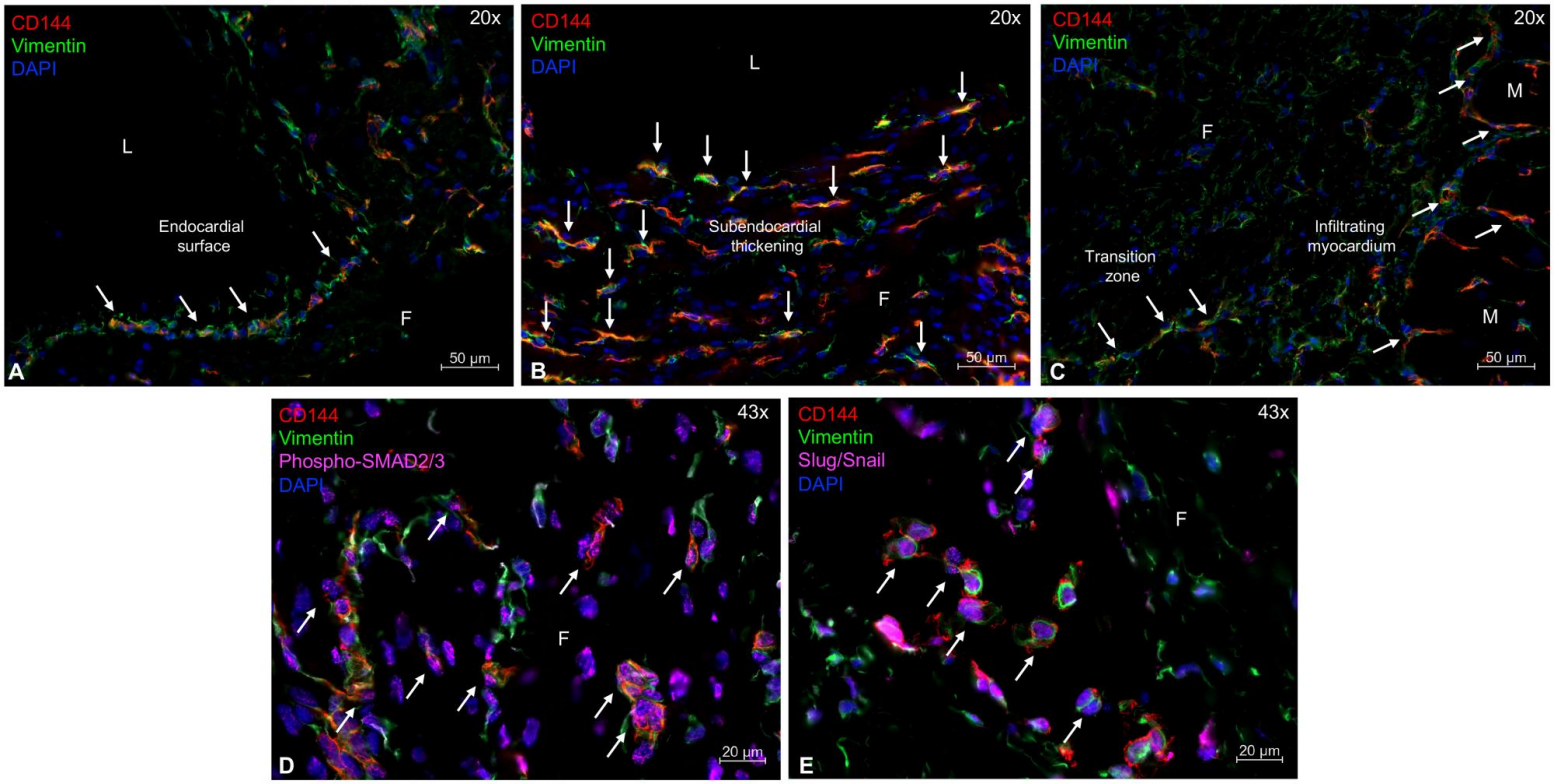
**Immunohistochemical staining demonstrates the presence of active TGF-β-mediated EndMT in PA/cPS-IVS.** Double-positive cells (white arrows) co-expressing the endothelial marker VE-cadherin (red) and the mesenchymal marker vimentin (green) were observed at the (A) endocardial surface, within (B) subendocardial tissue, and (C) infiltrating the underlying myocardium. (D) These regions also exhibited VE-cadherin/vimentin co-expression with nuclear pSMAD2/3 (pink), indicating partly TGF-β signalling. (E) Nuclear co-localization with Slug/Snail (pink) further confirmed active EndMT. Nuclei are counterstained with DAPI (blue). Abbreviations: EndMT, endothelial-to-mesenchymal transition; F, fibroelastic tissue; L, lumen; M, myocardium; PA/cPS-IVS, pulmonary atresia/congenital pulmonary stenosis with intact ventricular septum; TGF-β, transforming growth factor-beta.

In all patients, immunohistochemical staining demonstrated double positivity of EECs for VE-cadherin and vimentin within the subendocardium and the infiltrated myocardium (**Figure 3A-C**). A total of 67.1% (SD: 15.1) of EECs co-expressed both markers. There was also no age difference observed (data not shown). Additionally, double-positive EECs showed nuclear localization of phospho-SMAD2/3, indicative of partly TGF-β-driven signalling, and nuclear co-expression of the transcription factor Slug/Snail, consistent with active EndMT (**Figure 3D and E**). Healthy RV tissue showed no evidence of EndMT or activation of its underlying signalling pathways (**Figure S2**).

**Figure 4.**
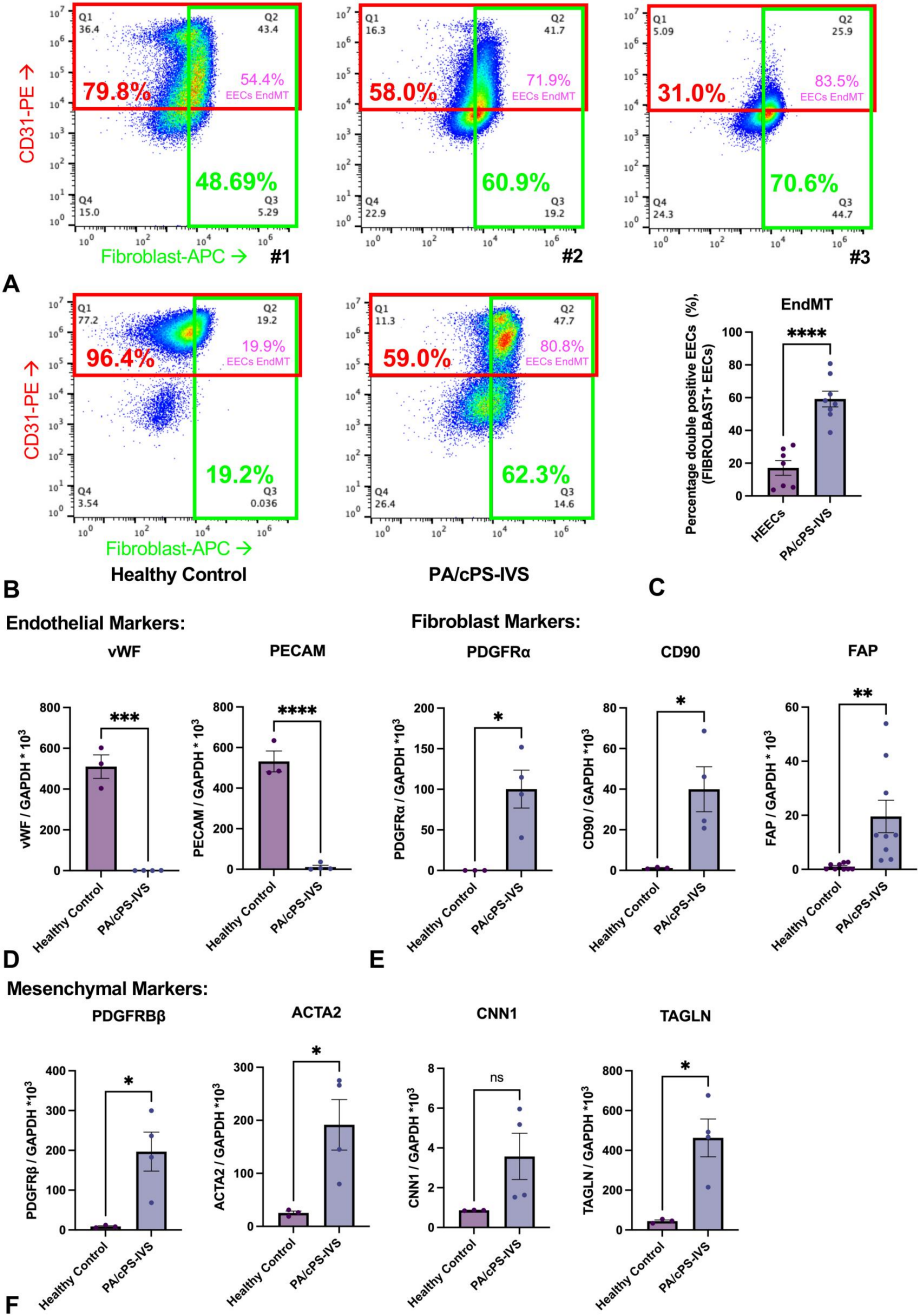
**PA/cPS-IVS-EECs exhibited fibrogenic activation at both the protein and transcriptomic levels.** (A) Flow cytometry: analysis of PA/cPS-IVS tissue assessing CD31 (endothelial) and a fibroblast (mesenchymal) marker expression. (B) Flow cytometry: Analysis of magnetically purified EECs, showing an increased CD31-positive/fibroblast-marker-positive double-positive EEC-fraction in PA/cPS-IVS. (C) Summary graph: flow cytometry reveals significantly higher proportion of double-positive EECs in PA/cPS-IVS than in controls. (D) Endothelial markers: qRT-PCR quantification of PECAM1 and VWF gene expression. (E) Fibroblast markers: qRT-PCR quantification of PDGFRα, CD90, and FAP gene expression. (F) Mesenchymal markers: qRT-PCR quantification of PDGFRβ, ACTA2, CNN1, and TAGLN gene expression. Unpaired t-test analysis. Graphs represent the mean with SE. Scatter dot plots are biological replicates (N). *P < .05, **P < .01, ***P < .001, ****P < .0001. Abbreviations: ACTA2, Actin alpha 2, smooth muscle; CD31, cluster of differentiation 31; CD90, cluster of differentiation 90; CNN1, calponin 1; EECs, endocardial endothelial cells; EndMT, endothelial-to-mesenchymal transition; FAP, fibroblast activation protein; HEECs, human endocardial endothelial cells; PA/cPS-IVS, pulmonary atresia/congenital pulmonary stenosis with intact ventricular septum; PDGFRα, platelet-derived growth factor receptor alpha; PDGFRβ, platelet-derived growth factor receptor beta; PECAM1, platelet endothelial cell adhesion molecule 1; qRT-PCR, quantitative reverse transcription polymerase chain reaction; TAGLN, transgelin; VWF, von Willebrand factor.

Flow cytometry analysis supported the immunohistochemical findings with 18.1% (SD: 15.6) CD31-positive, but 65.5% (SD: 26.7) fibroblast-marker-positive cells in the analysed isolated tissue. Notably, 77.1% (SD: 20.0) of all EECs were undergoing EndMT. Representative flow cytometry plots are depicted in **Figure 4A**. After 48 hours under pathological static conditions, purified PA/cPS-IVS-EECs had a significantly higher proportion of double-positive EECs compared with HEECs, indicative of EndMT: 59.2% (SD: 13.5) vs 17.1% (SD: 11.8) (*P* < .001; **Figure 4B and C**). Additionally, we provide supporting data that the fibrogenic activation of EECs through EndMT is endocardial cell-specific (**Figure S3**) and partly responsive to TGFβ1-protein stimulation (**Figure S4**).

Furthermore, at the gene expression levels, PA/cPS-IVS-EECs exhibited a marked reduction in the expression of endothelial markers, including platelet endothelial cell adhesion molecule 1 (PECAM1, *P* ≤ .001) and von Willebrand factor (*P* = .001) compared with HEECs (**Figure 4D**). Analysis of fibroblast-associated genes revealed a significant upregulation of platelet-derived growth factor receptor alpha (PDGFRα, *P* = .015) and cluster of differentiation 90 (CD90, *P* = .032) in PA/cPS-IVS-EECs, and also significant expression of fibroblast activation protein, suggesting a transitional phenotype towards activated stromal cells and indicating a progressive fibrotic state (*P* = .007; **Figure 4E**).^26^ Finally, expression of mesenchymal markers was significantly increased, including platelet-derived growth factor receptor beta (PDGFRβ, *P* = .023), actin alpha 2, smooth muscle (ACTA2, *P* = .032), and transgelin (TAGLN, *P* = .014), whereas expression of calponin 1 (CNN1, *P* = .106) remained unchanged (**Figure 4F**).

### PA/cPS-IVS-EECs lose their endothelial phenotype

**Figure 5.**
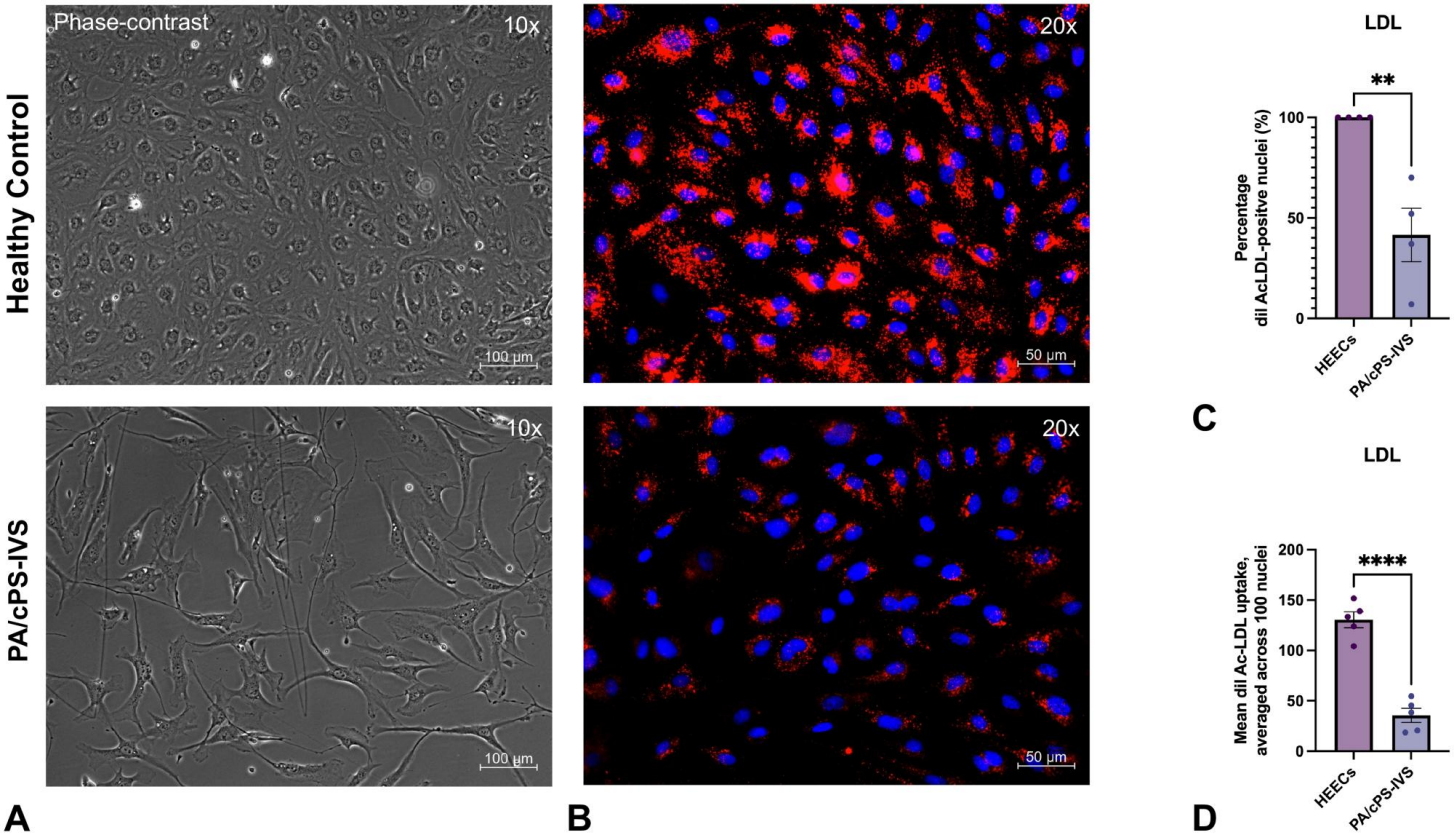
**PA/cPS-IVS-EECs loose their endothelial phenotype and associated function.** (A) Morphology (phase-contrast): HEECs displayed the typical cobblestone-like monolayer of endothelial cells, whereas PA/cPS-IVS EECs demonstrated a spindle-shaped, mesenchymal-like morphology. (B) LDL uptake: HEECs showed a robust DiI-Ac-LDL uptake (red). In contrast, PA/cPS-IVS-EECs demonstrated markedly reduced LDL uptake and fewer DiI-Ac-LDL-positive (red) nuclei (blue). (C-D) Summary graphs: both the percentage of DiI-Ac-LDL-positive nuclei and the mean DiI-Ac-LDL signal intensity per nucleus were significantly reduced in PA/cPS-IVS-EECs vs controls. Unpaired *t*-test analysis. Graphs represent the mean with SE. Scatter dot plots are biological replicates (N). **P < .01, ***P < .001. Abbreviations: DiI-Ac-LDL, Acetylated low-density lipoprotein label;ed with 1,1′-dioctadecyl-3,3,3′,3′-tetramethylindocarbocyanine perchlorate; EECs, endocardial endothelial cells; HEECs, human endocardial endothelial cells; PA/cPS-IVS, pulmonary atresia/critical pulmonary stenosis with intact ventricular septum.

PA/cPS-IVS-EECs displayed a spindle-shaped, mesenchymal-like morphology, unlike the cobblestone-like monolayer of HEECs (**Figure  5A**). After 48 hours under pathological static conditions, the efficiency of LDL uptake was significantly reduced in PA/cPS-IVS-EECs compared to HEECs with a marked decrease in LDL-positive cells (*P* = .005) and a reduction in the mean LDL uptake across 100 positive nuclei (*P* < .001) (**Figure  5B-D**). This is indicative of a significant loss of the endothelial phenotype and function.

### Pathological flow induces EndMT

**Figure 6.**
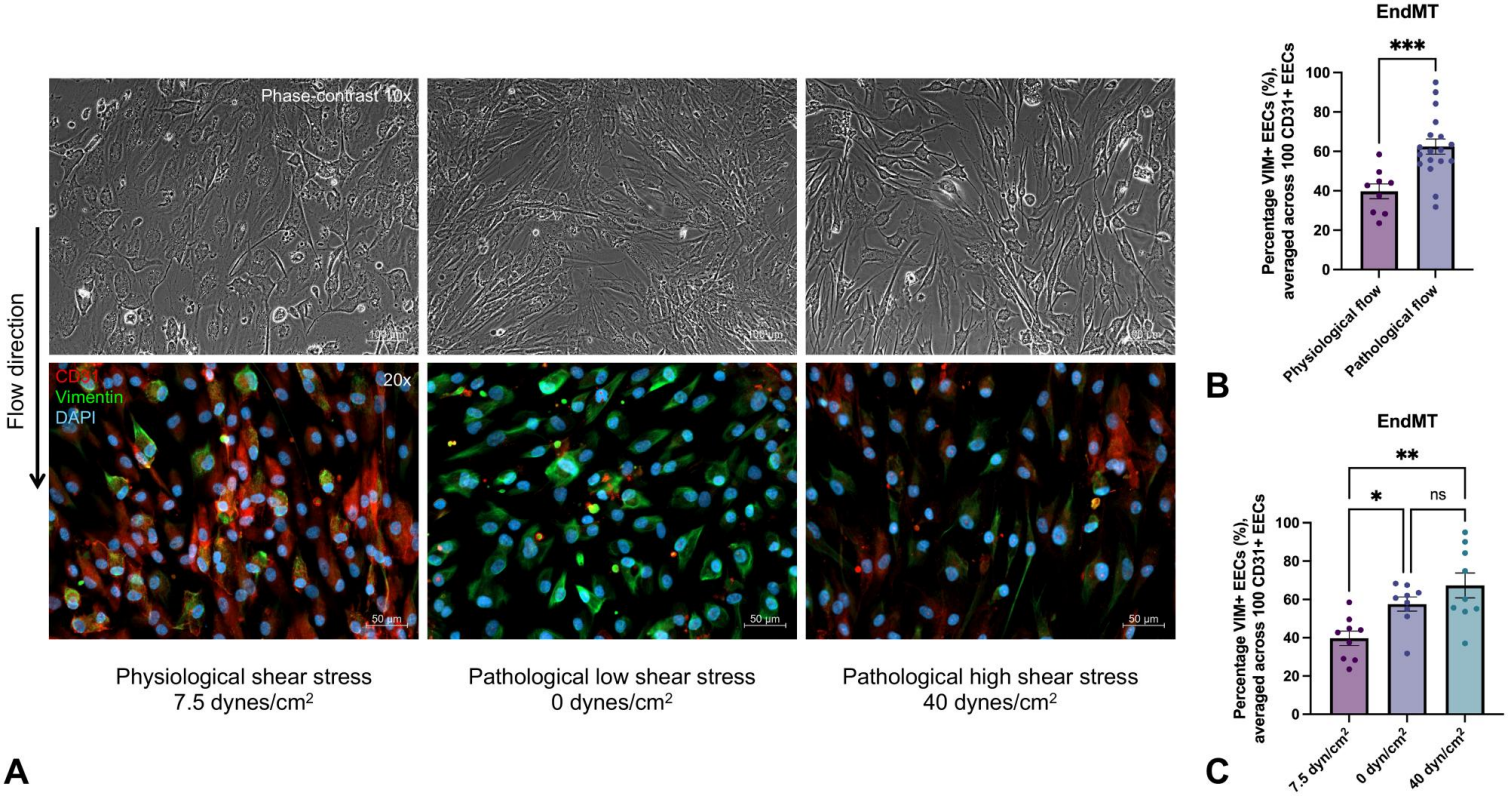
**Fibrogenic activation of PA/cPS-IVS-EECs through EndMT following 48 hours of exposure to physiological vs pathological shear stress.** (A) Morphology (upper panel, phase-contrast): Under physiological flow, PA/cPS-IVS EECs aligned in the direction of flow. Pathological flow disrupted cell-cell contacts and induced a spindle-shaped, mesenchymal-like morphology. Immunocytochemistry (lower panel): pathological flow reduces CD31 (red) and increases vimentin (green) expression compared with physiological flow. (B) Summary graph: pathological flow significantly increases double-positive EECs. (C) Summary graph: pathological no-flow (static) and high-flow conditions do not differ in their effect on double-positive EECs. Unpaired *t*-test analysis (B) and one-way ANOVA with Bonferroni’s post-test analysis (C). Graphs represent the mean with SE. Scatter dot plots are biological replicates (N). *P < .05, **P < .01. Abbreviations: CD31, cluster of differentiation 31; EECs, endocardial endothelial cells; EndMT, endothelial-to-mesenchymal transition; PA/cPS-IVS, pulmonary atresia/critical pulmonary stenosis with intact ventricular septum.

PA/cPS-IVS-EECs under physiological flow form a continuous monolayer with cobblestone-like appearance aligned in the direction of flow. In contrast, cells exposed to pathological shear stress exhibit disrupted cell-cell contacts and a spindle-shaped, mesenchymal-like morphology (**Figure 6**). Pathological flow conditions mimicking the human disease, whether due to flow stagnation, i.e., reduced valve inflow (0 dynes/cm^2^), or flow acceleration, that is, high velocity jets due to valve stenosis (40 dynes/cm^2^), led to a significant increase in double-positive EECs compared to physiological flow (7.5 dynes/cm^2^), rising from 39.7% (SD: 11.2) to 62.4% (SD: 16.2) (*P* < .001) (**Figure 6A-C**, **Figure S3**). In detail, PA/cPS-IVS-EECs under physiological flow showed significantly fewer EECs undergoing EndMT compared to both no-flow and pathological high-flow conditions: 39.7% (SD: 11.2) vs 57.6% (SD: 11.1) and 67.3% (SD: 19.6) (*P* = .002). However, when comparing different pathological flow types, there was no difference observed between no flow (0 dynes/cm^2^) and pathological high flow (40 dynes/cm^2^) (*P* = .348). Compared with HEECs, PA/cPS-IVS-EECs demonstrated a distinct vulnerability to fibrogenic activation under pathological flow conditions (**Figure S5**).

## Discussion

This study demonstrates that fibroelastic remodelling occurs in the RV in the setting of PA/cPS-IVS, as evidenced by hallmarks of EndMT: (1) protein expression changes, including reduction of endothelial and gain of mesenchymal markers, (2) upregulation of transcription factor Slug/Snail, (3) morphological shift, with reduction of cobblestone-like monolayer integrity and transition to a spindle-shaped, fibroblast-like phenotype, and (4) functional changes, reflected by impaired endothelial function. Furthermore, our results indicate that EndMT is at least partly mediated through TGF-β-signalling. Histological and immunohistochemical evidence confirms that the fibroelastic remodelling in PA/cPS-IVS is analogous to EFE, characterized by infiltrative growth patterns into the underlying myocardium, resulting in diastolic dysfunction.

In our patient cohort, fibroelastic remodelling was present in at least 60.7% (17/28) of patients with PA/cPS-IVS. Interestingly, this finding aligns with the reported prevalence of fibroelastic remodelling in the haemodynamically equivalent left-sided disease.^27^ However, its prevalence may depend on the underlying anatomic subtype. As our study focuses on a selected subgroup of the overall population, conclusions regarding the true prevalence of this entity in the full PA/cPS-IVS spectrum should therefore be interpreted with caution. All patients presented with flow disturbances across the PV and/or TV, resulting from jets or to-and-fro flow across stenotic or incompetent tricuspid or PVs. Tissue samples showed localized fibrogenic activation of EECs in the RV. This aligns with our recent findings that flow disturbances across abnormal muscle bundles and ventricular septal defects in the RVOT also promote fibroelastic remodelling.^25^ Histological analysis revealed fibroelastic remodelling with distinct layers of collagen and elastic fibres with infiltrative growth into the underlying myocardium, without age-related differences, consistent with our previous findings in the left-sided haemodynamic equivalent disease.^18^^,^^21^^,^^23^

While we considered flow disturbances as key contributors to disease development and progression, we also observed that several patients had RV pressures at or above systemic levels. Such pressure overload can generate mechanical stretch, another well-established inducer of EndMT, as demonstrated in our previous work.^21^ Accordingly, the nuclear co-localization of phosphorylated SMAD2/3 in our samples may also be driven by mechanical stretch resulting from ventricular pressure overload, rather than only flow disturbances, further underscoring the complexity of this pathology. However, to the best of our knowledge, this is the first study to demonstrate a disease-driving role of, at least partly, canonical TGF-β-signalling in this entity.

Vascular endothelial cells normally adapt to mechanical stimuli through dynamic changes in shape and function; however, pathological flow can disrupt this homeostasis and trigger maladaptive plasticity, as observed in EndMT.^28^^,^^29^ In support, our cell culture experiments show that PA/cPS-IVS-derived EECs in the RV exhibit a distinct predisposition to fibrogenic activation in response to disturbed flow, likely mediated by TGF-β-activation. This behaviour appears to be a distinct characteristic of pathologically impaired EECs in PA/cPS-IVS. When exposed to the same pathological stressors, HEECs exhibit some EndMT, but without differences across flow conditions. In contrast, PA/cPS-IVS-EECs show significant changes, even under physiological flow conditions, highlighting their distinct predisposition to EndMT and vulnerability to pathological flow. It is important to note that our model cannot replicate the *in vivo* multidimensional shear stress (e.g., turbulent flow) and biomechanical forces (e.g., pulsatile blood flow) and thus mimics the human disease to a limited extent.

Fibroelastic remodelling increases ventricular stiffness and contributes to diastolic dysfunction.^14^^,^^15^^,^^22^^,^^23^^,^^30^ This is the first study to comprehensively link endocardial fibroelastic remodelling with diastolic dysfunction in PA/cPS-IVS. All patients showed reduced diastolic RV compliance as evidenced by elevated filling pressures, suggesting a pathophysiological role for fibroelastic remodelling in RV diastolic dysfunction. Normal mPAP and PVRI indicated that the dysfunction resulted from a small, stiff ventricle rather than increased afterload. Elevated RA pressures with prominent a-waves further reflected impaired filling, however, likely exacerbated by the high prevalence of tricuspid obstruction in this cohort.

In contrast to previous reports of a 55.4% recurrence rate at a median of 26.4 months (IQR: 13.2-44.4),^14^ our cohort showed slightly more favourable outcomes with only 38.5% (5/13) occurring at 27.3 months (IQR: 10.3-48.7), however, with a median follow-up period of only 12.4 months (IQR: 1.9-33.5). Interestingly, 80.0% (4/5) of patients with noted recurrences demonstrated haemodynamically significant flow disturbances due to stenotic or incompetent TV, as evidenced by a tricuspid inflow jet detected on echocardiography at last follow-up. This observation suggests that persistent flow disturbances may, at least in part, contribute to the recurrence of fibroelastic remodelling. While long-term follow-up of our cohort is not yet available, addressing the underlying flow disturbances may help prevent localized fibroelastic remodelling in the RV. Nevertheless, one patient ultimately required Fontan palliation during follow-up due to a lack of RV growth and progressive dysfunction. In general, the more favourable outcomes may also reflect the advantageous baseline characteristics of our cohort, including membranous PA/cPS-IVS without RV-dependent coronary circulation and predominantly moderate RV size, allowing for ventricular recruitment. In addition, the presence of only mild-to-moderate TV hypoplasia is likely beneficial, as less severe TV hypoplasia has been shown to predict the feasibility of successful two-ventricle repair.^31^^,^^32^ Collectively, these features are generally associated with improved RV development and long-term prognosis.^10^^,^^33^

Although it has recently been reported that improved intracardiac blood flow promotes favourable RV remodelling in PA/cPS-IVS, there remains a need to thoroughly investigate diastolic function and fibrosis at the endocardial level to fully characterize the potential remodelling response in this population.^34^ Despite these promising results, the notable recurrence rates suggest that surgical resection with restoring a more physiological flow profile alone may not be sufficient, underscoring the need for adjunctive therapies. The differentially expressed markers identified in this study may serve as potential therapeutic targets for individualized treatment and warrant future investigation.

## Conclusions

Our study shows that, in patients with PA/cPS-IVS, restrictive RV physiology and diastolic dysfunction are associated with fibroelastic remodelling in the subendocardium. Pathological intracardiac flow disturbances promote localized fibrogenic activation of EECs via EndMT in the RV of patients with PA/cPS-IVS. This mechanistic link is consistent with prior studies demonstrating that abnormal flow and mechanical stretch can trigger EndMT in congenital heart disease. These results suggest that targeting fibrogenic pathways may represent a promising therapeutic strategy in PA/cPS-IVS.

### Limitations

Our findings apply to a subgroup of patients with PA/cPS-IVS with mild-to-moderate disease severity. As this study is observational, causality cannot be definitively established, and the conclusions drawn from the statistical significance should be interpreted carefully. Further mechanistic and interventional studies in larger, prospective cohorts with longer follow-up are warranted to validate these findings and evaluate therapeutic interventions. However, overall, these data advance our understanding of the pathogenesis of fibroelastic remodelling in PA/cPS-IVS and highlight the need for translational research to develop targeted therapies.

## Supplementary Material

ezag143_Supplementary_Data

## Data Availability

Supporting data are available from the corresponding author upon request.

## Abbreviations

1.5V: one-and-a-half ventricular
2V: biventricular
Ac-LDL: acetylated low-density lipoprotein labelled with 1,1'-dioctadecyl- 3,3,3',3'-tetramethylindocarbocyanine perchlorate
ACTA2: actin alpha 2, smooth muscle
CD31: cluster of differentiation 31
CD90: cluster of differentiation
CNN1: calponin 1
ECGM-2: endothelial cell growth medium-2
EECs: endocardial endothelial cells
EFE: endocardial fibroelastosis
EndMT: endothelial-to-mesenchymal transition
EVG: elastin van Gieson
FAP: fibroblast activation protein
H&E: hematoxylin and eosin
HEECs: healthy endocardial endothelial cells
LV: left ventricular
mPAP: mean pulmonary artery pressure
MT: Masson’s trichrome
PA/cPS-IVS: pulmonary atresia/critical pulmonary stenosis with intact ventricular septum
PDGFRα: platelet-derived growth factor receptor alpha
PDGFRβ: platelet-derived growth factor receptor beta
PECAM1: platelet endothelial cell adhesion molecule 1
pSMAD2/3: phosphorylated suppressor of mothers against decapentaplegic 2/3
PVRI: pulmonary vascular resistance index
RA: right atrium
RVOT: right ventricular outflow tract
TGF-β: transforming growth factor-beta
TAGLN: transgelin
VE-Cadherin: vascular endothelial cadherin
