# Supplementary material for: Fibroelastic Remodelling of the Endocardium on the Right Side of the Heart: Endothelial-to-Mesenchymal Transition in Pulmonary Atresia With Intact Ventricular Septum
Source: Eur J Cardiothorac Surg. 2026 Apr 3;68(4):ezag143. doi: 10.1093/ejcts/ezag143 (PMC13094542; doi:10.1093/ejcts/ezag143)
Supplement: ezag143_Supplementary_Data [file ezag143_supplementary_data.zip › Supplementary_Table_S1-2.pdf]

Supplementary Table S1: Preoperative morphological and hemodynamic characteristics of included patients with PA/cPS-IVS

Patient	Age (years)	Cardiac anatomy	RV morphologic features and function	RVEDP (mmHg)	RV lateral s' velocity (cm/s), z-score	RV/systemic pressure ratio	Tricuspid anulus diameter (mm), (z-score)	Pulmonary anulus diameter (mm), (z-score)	PA pressure (mmHg)	Pulmonary vascular resistance (iWU)	RA pressure (mmHg)	A-wave (mmHg)	Surgery at the time of fibrous tissue resection	Circulation after fibrous tissue resection surgery
1	0.96	PA-IVS with subvalvar muscular narrowing, ASD, PFO, PDA, RV-CAF	Mildly hypoplastic TV; Moderately hypoplastic RV with severe hypertrophy and subvalvar narrowing; Fistulous connections from RV to LAD; Mild systolic dysfunction	12	Not measured	Not measured	9.5 (-2.6)	10.7 (-0.9)	12	2	9	12	mBTTS takedown, BDG, RVOT reconstruction (14mm RV-PA conduit), fibrous tissue resection, TV repair, PFO restriction	1.5V
2	4.34	Near membranous PA-IVS, ASD, PDA	Mildly hypoplastic TV; Hypertrophied, muscle-bound RV	14	5.8; (-3.0)	0.26; RV 20 mmHg against systolic pressure 76 mmHg	13.7 (-2.3)	16.2 (-5.67)	13.5	2.6	13.5	17.5	TV repair, fibrous tissue resection, PV replacement (19 mm porcine valve), fenestrated ASD closure	1.5V
3	5.11	Membranous PA-IVS, ASD, PFO, anomalous origin of RCA from the left sinus of valsalva	Moderately hypoplastic TV that severely narrows toward the septum tantamount to a parachute TV; Severely hypertrophed, moderately hypoplastic RV; Moderate dysfunction	Not measured	6.0; (-3.0)	Not measured	11.0 (-3.2)	Atretic	12	1.9	7	9	RV recruitment (opening of TV/PV, TV repair, thinning PV leaflets, division muscle bundles, fibrous tissue resection), fenestrated patch between RPA and MPA, fenestrated ASD closure	1.5V

4	9.9	Membranous PA-IVS, ASD	Moderately hypoplastic TV with severely reduced orifice area, and trivial inflow; Mild-to-moderately hypoplastic and severely hypertrophied RV with severely increased trabeculations; Moderate-to-severe dysfunction	11	Not measured	1.27 RV 115 mmHg against systolic pressure 90 mmHg	12.7 (-3.3)	Atretic	17.5	3.3	13	Not measured	Fontan takedown, RV recruitment (RV muscle bundle/fibrous tissue resection, RVOT reconstruction (Gore-Tex monocusp/ transannular patch), TV repair), fenestrated membrane between central PA and BDG, fenestrated ASD closure	1.5V
5	3.45	Membranous PA-IVS, ASD, RV-CAF	Severely hypoplastic TV; Severely hypoplastic RV with increased trabeculations; Moderate-to-severe systolic dysfunction	13.5	4.1; (-3.8)	1.83 RV 165 mmHg against systolic pressure 90 mmHg	5.9 (-5.2)	Atretic	15	1.6	8	12.5	RV recruitment (RV muscle bundle/fibrous tissue resection, RVOT reconstruction (transannular patch), TV repair), septation between MPA and RPA, fenestrated ASD closure	1.5V
6	4.39	Membranous PA-IVS, ASD, MR	Moderately hypoplastic TV; Moderate-to-severly hypoplastic and hypertrophied RV with prominent muscle bundles; Severe systolic dysfunction	9	3.7; (-4.1)	1.36 RV 105 mmHg against systolic pressure 77 mmHg	11.9 (-2.5)	Atretic	14	1.1	8	Not measured	RV recruitment (RV muscle bundle/fibrous tissue resection, RVOT reconstruction (transannular patch)), fenestrated membrane between RPA and LPA, fenestrated ASD closure	1.5V

7	0.96	Critical PS-IVS (near atretic), PFO, RV-CAF	Subvalvar and valvar PS; Moderately hypoplastic TV; moderately hypertrophied, heavily muscle-bound RV; Severe systlic dysfunction	12	1.0; (-4.6)	1.0<br>RV 74mmHg against systolic pressure 74 mmHg	9.3 (-2.8)	7.3 (-2.3)	13	1.7	7	13	RV recruitment (division muscle bundles, fibrous tissue resection, RVOT reconstruction (transannular patch), PV reconstruction (monocusp leaflet), mobilization of TV papillary muscles), PDA ligation, fenestrated ASD closure	2V
8	0.61	Membranous PA-IVS, ASD	Severely hypoplastic TV with extremely restricted leaflet excursion; Moderate-to-severely hypoplastic and hypertrophied RV	Not measured	Not measured	Not measured	7.8 (-4.4)	9.9 (-1.0)	13	0.8	8	13	RV recruitment (RV muscle bundles/fibrous tissue resection, RVOT incision, TV repair), BDG, PDA stent removal, LPA plasty, narrowing ASD, RA cryomaze	1.5V
9	6.74	Membranous PA-IVS, ASD, bicuspid AoV, separate origin of conal branch adjacent to RCA origin, LCA from left posterior sinus, RV-CAF	Severely hypoplastic TV; Severely hypoplastic, hypertrophied, nearly apex-forming muscle-bound RV with increased trabeculations; Severe systolic dysfunction	12	Not measured	1.40<br>RV 115mmHg against systolic pressure 82 mmHg	7.0 (-5.4)	Atretic	13	1.7	9	Not measured	RV recruitment (RV muscle bundle/fibrous tissue resection, RVOT reconstruction (transannular patch), TV repair), fenestrated PA membrane, fenestrated ASD closure	1.5V

10	1.02	Membranous PA-IVS,near total infindibular obstruction due to muscular narrowing, PFO, RV-CAF, RCA arises leftward from aorta superior to the sino-tubular junction, LUPV atresia, LLPV stenosis	Mildly hypoplastic TV; Moderate-severe hypoplastic and hypertrophied RV; Moderate systolic dysfunction	16	8.4; -1.0	0.63 RV 45mmHg against systolic pressure 72 mmHg	8.7 (-2.7)	13.8 (-5.08)	25	3.6	8	14	RV recruitment (RV muscle bundle/fibrous tissue resection, RVOT reconstruction (transannular patch), TV repair), PDA stent resection, fenestrated ASD closure	2V
11	2.49	Membranous PA-IVS, ASD, VSD, Double outlet right atrium, RV-CAF, RCA atresia	Moderately hypoplastic TV; moderately hypoplastic, hypertrophed non-apex-forming RV	15	7.0; -2.2	0.41 RV 31mmHg against systolic pressure 74 mmHg	8.6 (-3.6)	9.7 (-1.6)	18	2.6	10	15	RVOT reconstruction (14 mm RV-PA conduit), fibrous tissue resection, TV repair, fenestrated ASD closure	1.5V
12	0.38	Membranous PA-IVS, PDA	Normal TV dimension with limited diastolic motion; Mildly hypoplastic, hypertrophied muscle-bound RV; Mild systolic dysfunction	12	9.4; -0.2	1.0 RV 67mmHg against systolic pressure 67 mmHg	14.0 (0.4)	10.7 (-0.3)	13	2.6	6	16	RV recruitment (RVOT reconstruction (PV monocusp), RV muscle bundle/fibrous tissue resection, TV repair), fenestrated ASD closure, PDA division	2V
13	5.13	Membranous PA-IVS, PFO, RV-CAF	Mildly hypoplastic TV with limited motion and small orifice; mildly hypertrophied RV; Mild systolic dysfunction	10	5.8; -3.1	0.32 RV 29mmHg against systolic pressure 92 mmHg	12.1 (-2.7)	10.2 (-2.2)	14	2.3	10	Not measured	PV replacement (19mm epic valve), TV repair, fibrous tissue resection, fenestrated	1.5V

AoV = Aortic Valve, ASD = Atrial Septal Defect, BDG = Bidirectional Glenn shunt, cPS-IVS = Critical Pulmonary Stenosis with Intact Ventricular Septum, LAD = Left Anterior Descending Coronary Artery, LCA = Left Coronary Artery, LLPV = Left Lower Pulmonary Vein, LPA = Left Pulmonary Artery, LUPV = Left Upper Pulmonary Vein, MPA = Main Pulmonary Artery, MR = Mitral Regurgitation, mBTTS = Modified Blalock–Taussig–Thomas Shunt, PA = Pulmonary Artery, PA-IVS = Pulmonary Atresia with Intact Ventricular Septum, PDA = Patent Ductus Arteriosus, PFO = Patent Foramen Ovale, PV = Pulmonary Valve, RA = Right Atrium, RCA = Right Coronary Artery, RPA = Right Pulmonary Artery, RV = Right Ventricle, RV-CAF = Right Ventricle to Coronary Artery Fistula, RVEDP = Right Ventricular End-Diastolic Pressure, RVOT = Right Ventricular Outflow Tract, RV-PA conduit = Right Ventricle to Pulmonary Artery conduit, TV = Tricuspid Valve, VSD = Ventricular Septal Defect, 1.5V = One-and-a-half ventricle circulation, 2V = Biventricular circulation.

Supplementary Table S2: Detailed follow-up information

Patient	Hemodynamic relevant flow disturbances caused by stenotic/incompetent vales, at last follow up (assessed by echocardiography)	Re-intervention/-operation	Time, post-operative (months)	Recurrence fibroelastic tissue	Time, post-operative (months)	Follow-up time (months)
1	TS/TR	0		0		33.64
2	TR/TS with turbulences (jet), PR/PS (jet)	0		1	53.48	53.54
3	PS, TR (mild flow acceleration)	0		0		12.36
4	TS/TR (jet)	0		1	43.87	43.87
5	None	0		0		19.77
6*[1]	TR	1	33.2	1	11.84	33.34
7	PR, TR (broad inflow jet)	0		0		0.16
8*[2]	TR (inflow jet)	1	13.16	1	27.25	27.51
9	TS	0		0		0.43
10	PR, TR (inflow jet)	0		1	8.82	8.89
11	TS (reduced flow)	0		0		0.16
12	PR, thickened tricuspid valve leaflet with restrictive opening (trivial TS/TR)	0		0		5.18
13	TR	0		0		3.31

PR = Pulmonary regurgitation, PS = Pulmonary stenosis, TR = Tricuspid regurgitation, TS = Tricuspid stenosis

*[1] 33.2 months post-repair, patient no. 6 was converted from 1.5-ventricle to Fontan circulation due to the absence of RV growth and progressive RV dysfunction.

*[2] Patient no. 08 required two re-operations: the first at 13.2 months post-repair, which included re-resection of fibrous tissue, TV repair, and insertion of an RV to PA conduit, and the second at 27.3 months, involving re-resection of fibrous tissue, TV repair, pulmonary valve replacement, and Glenn take-down.
