# Supplementary material for: Fibroelastic Remodelling of the Endocardium on the Right Side of the Heart: Endothelial-to-Mesenchymal Transition in Pulmonary Atresia With Intact Ventricular Septum
Source: Eur J Cardiothorac Surg. 2026 Apr 3;68(4):ezag143. doi: 10.1093/ejcts/ezag143 (PMC13094542; doi:10.1093/ejcts/ezag143)
Supplement: ezag143_Supplementary_Data [file ezag143_supplementary_data.zip › Supplementary File S1.pdf]

1. Ethics Statement

This research project was approved by the institutional review board (IRB) of Boston Children's Hospital (Protocol numbers: P0038762 and P00026224) and conducted in compliance with its ethical standards. Following approval, resected tissue was obtained directly from the operating room (OR) and processed in the laboratory for downstream applications.

2. Patients

This study included patients diagnosed with PA/cPS-IVS who underwent staged ventricular rehabilitation surgery aiming at the preservation of the RV for 1.5 ventricular (1.5V) or biventricular (2V) circulation from March 2021 to July 2025 at Boston Children's Hospital, during which tissue specimens were resected. Anthropometric and clinical data, procedural details, and short-term follow-up information were obtained by reviewing medical and surgical records. All patients underwent at least one pre- and postoperative echocardiogram, along with a preoperative diagnostic catheterization. For comparison, healthy RV tissue from postmortem donors (N = 3) were included as controls.

3. Histologic Assessment

Tissue samples were promptly embedded in an optimal cutting temperature compound, snap-frozen, and stored at –80°C until sectioning and processing for microscopic analysis. Overall tissue morphology was evaluated using Hematoxylin and Eosin (H&E) staining. Collagen content was visualized with Masson's Trichrome (MT) staining, while elastin deposition was analyzed using Elastica van Gieson (EVG) staining. Disproportionate accumulation of tissue characterized by low cellular content, and organized layers of collagen and elastin fibers was classified as fibroelastic tissue, resembling endocardial fibroelastosis (EFE). Tissue sections were imaged in their entirety using a Zeiss Axio Observer Z1 inverted microscope with ZEN 3.6 (blue edition) software at 20× magnification. Individual 20× images were subsequently stitched into a single composite image using Affinity Photo (Version 1.10.8). Collagen content was quantified by

morphometric differentiation of blue-stained collagen fibers from red-stained cellular components, and elastin content was quantified by differentiation of black elastin fibers from red/yellow cellular components, across the entire tissue section using ImageJ (Java 8). The percentage area of fibrosis and elastin deposition was calculated by dividing the summed fibrotic or elastic areas across all sections by the total tissue area.

4. Immunohistochemistry (IHC)

The tissue was fixed in 4% paraformaldehyde (PFA, #15714S, Electron Microscopy Sciences) for 15 minutes at room temperature (RT), washed with phosphate-buffered saline (PBS, #10010023, Gibco), and blocked in blocking buffer for 1 hour at RT. Then primary antibodies were applied overnight at 4–8 °C in dilution buffer (PBS with 5% normal goat serum (NGS, #31872, Invitrogen) 0.3% Triton-X100 (#X100, Sigma Aldrich), and 0.1% bovine serum albumin (BSA, #A7030-10G, Sigma Aldrich)). The following day, the slides were washed with PBS and incubated for 90 minutes at RT with secondary antibodies diluted in dilution buffer. Finally, three additional washes with PBS were followed by 4',6-diamidino-2-phenylindole (DAPI, #D1306, Invitrogen) nuclear counterstaining. The tissue was stained with the endothelial marker CD144 (1:100, #14-1449-82, Invitrogen) and the mesenchymal marker vimentin (1:100, #MA5-11883-A488, Invitrogen). Co-expression of these markers was considered indicative of EndMT. To evaluate the involvement of a TGFB-driven pathway and determine whether active EndMT at the time of tissue resection was present, we stained for the endothelial marker CD144 and phosphorylated SMAD2/3 (pSMAD2/3, 1:50, #PA5-110155, Invitrogen), or transcription factors Slug/Snail (1:50, #ab85936, Abcam), respectively. Nuclear co-localization of pSMAD2/3 or Slug/Snail indicated an underlying TGFB-driven pathway, and active EndMT. For secondary antibodies we used Goat anti-Rabbit Alexa Fluor Plus 647 (1:200, #A32733, Invitrogen), and Goat anti-Mouse Texas Red-X (1:200, #T-862, Invitrogen). Images were captured using a Zeiss Axio Observer Z1 inverted microscope with ZEN 3.6 (blue edition) software.

For each patient, 3-10 representative images containing areas with endothelial marker-positive cells were captured at 20× and 43× magnification. Cells were detected based on nuclear staining, and fluorescence intensity thresholds were applied to the Alexa Fluor 488 and Alexa Fluor 568 channels to identify marker-positive cells in tissue. Cells exceeding the threshold in both channels were classified as double positive. Quantification was performed using QuPath software (version 0.6.0).

5. Cell culture

Healthy endocardial endothelial cells (HEECs) were isolated from postmortem tissue obtained from cardiovascular-healthy donors (N = 3). The extracted tissue was rinsed with sterile PBS. The right ventricle (RV) was cut open to expose the cavity, placed face down in digestion buffer, and processed using the isolation procedures described below. PA/cPS-IVS-derived endocardial endothelial cells (PA/cPS-IVS-EECs) were isolated from the RV of pediatric patients diagnosed with PA/cPS-IVS and fibroelastic tissue. Tissue samples obtained directly from the OR were minced and placed in a 50 mL Falcon tube containing 23 mL of digestion buffer. The digestion buffer consisted of 18 mL Dulbecco's Modified Eagle Medium (DMEM, #11965092, Gibco), 2.5 mL D10 buffer (500 mL DMEM, 50 mL heat-inactivated fetal bovine serum (FBS, #FB-02, Omega Scientific), 5 mL Penicillin-Streptomycin-Glutamine (250 mg/L, #10378016, Gibco)), 2.5 mL calcium-magnesium ions (#C1016-100G, #M7506-500G, Sigma Aldrich), 100 mg Collagenase Type II (#7419, STEMCELL Technologies), 100 mg Collagenase Type A (#10103586001, Sigma Aldrich), and 100 mg Dispase II (#D4693-1G, Sigma Aldrich). Finally, 20 mL D10 buffer was added to the mixture. The tubes were placed on a rocking platform at 37°C for 2 hours. After digestion, the mixture was filtered through a 40 µm strainer and quenched with 10 mL of D10 buffer. The filtered suspension was centrifuged at 400g for 5 minutes at room temperature (RT), and the supernatant was completely aspirated. The resulting cell pellet was resuspended in 1 mL of Endothelial Cell Growth Medium-2 (ECGM-2, #CC-3162, Lonza Bioscience), containing supplements required for the growth of endothelial cells, as provided by the manufacturer. Besides, penicillin-streptomycin-glutamine (250 mg/L, #15140122, Gibco) was added to the medium. The

cell suspension was transferred onto a 1% gelatin-coated (#G1890, Gibco) culture dish. To prevent fibrogenic transformation via endothelial-to-mesenchymal transition (EndMT) into fibroblasts during cultivation, small molecule SB431542 that inhibits TGF-β signaling (6 mg/L, #130-106-543, Miltenyi Biotec) was added to the ECGM-2 medium. The cells were maintained in a humidified incubator at 37°C with 5% CO_2, and the medium was changed every other day until the EECs were reselected from the cultures.

PA/cPS-IVS-EECs were further purified using a CD144 antibody (#14-1449-82, Invitrogen) and Anti-Mouse IgG Microbeads (#130-048-402, Miltenyi Biotec) with MS Columns (#130-042-201, Miltenyi Biotec) placed in an OctoMACS separator (#130-042-108, Miltenyi Biotec). The CD144-labeled EECs were then plated onto 1% gelatin-coated petri dishes for downstream applications. HEECs did not require additional purification.

6. LDL-Assay

To evaluate endothelial cell function, specifically the capacity for acetylated low-density lipoprotein (Ac-LDL) uptake as a functional property of endothelial cells, an Ac-LDL uptake assay was performed. Approximately 20,000 EECs were seeded onto 12 mm round coverslips pre-coated with 1% gelatin in a 24-well plate and cultured to 30–40% confluency. Then, a washout phase was implemented by incubating the cells in ECGM-2 medium without TGF-β1 inhibitor for 24 hours. Following 48 hours, the cells were rinsed thrice with PBS and twice with serum-starved ECGM-2 medium lacking TGF-β1 inhibitor and FBS but supplemented with 0.3% BSA. DiI-labeled Ac-LDL (#L3484, Invitrogen) was added to the serum starvation medium to achieve a final working concentration of 10 µg/mL. The cells were incubated with this DiI AcLDL-containing medium for 90 minutes at 37°C. After incubation, the cells were washed three times with washing buffer (PBS containing 0.3% BSA) to remove excess DiI AcLDL. Finally, the cells were fixed with 4% PFA for 10 minutes, washed twice with PBS, and stored in buffer (PBS containing 0.1% BSA). Nuclei were counterstained with DAPI. For each patient, 10 images were randomly captured at 10× and 20×

magnification. Images were captured using a Zeiss Axio Observer Z1 inverted microscope with ZEN 3.6 (blue edition) software.

7. Shear Stress Experiments

PA/cPS-IVS-EECs were detached using 0.05% Trypsin-EDTA (#25300054, Gibco) and neutralized with Trypsin Neutralizer Solution (#R002100, Gibco), then diluted in fresh ECGM-2 to a concentration of 1.6×10^6 cells/mL. A 30 µL volume of this cell suspension (containing 48,000 cells) was pipetted into the reservoir of an ibidi slide (#80606-90, ibidi). After 2 hours in an incubator at 37°C to allow cell adhesion to the channel, 120 µL of additional EMG-2 was added. Experiments were initiated 24 hours after filling the reservoirs. Then cells were exposed to one of three conditions: low shear stress, no shear stress, or high shear stress (7.5, 0, or 40 dynes/cm^2) mimicking physiological flow (physiological), flow stagnation (pathological), or flow acceleration (pathological) for 48 hours. Cells were either subjected to 7.5 or 40 dynes/cm^2 using a commercially available flow pump system for cell culture under flow (ibidi, Fitchburg, WI) or kept in the incubator (0 dynes/cm^2), and the medium was replaced every 24 hours. After 48 hours, cells were fixed with 4% PFA at RT for 10 minutes, followed by two washes with washing buffer. Morphological changes were documented using a 10x inverted phase-contrast microscope.

Fixation of the cells was followed by blocking of non-specific binding using a washing buffer (PBS containing 5% NGS and 0.3% Triton X-100) at RT for 45 minutes. After removing the blocking buffer, primary antibodies in a dilution buffer (PBS with 1% BSA, 1% NGS, and 0.3% Tritton X-100) were applied and incubated overnight at 2-8°C. Cells were then washed twice with washing buffer and incubated with secondary antibodies in dilution buffer at RT in the dark for 1 hour. After two washes with washing buffer, the cells were incubated with DAPI solution at RT for 5 minutes, followed by a single wash with 1x PBS and 1x distilled water. Finally, one drop of ibidi mounting medium (#50001, ibidi) was applied to the reservoir, and the stained slides were stored at 2-8°C in the dark until imaging.

Primary antibodies included the endothelial marker CD31 (1:200, #ab32457, Abcam) and the mesenchymal marker vimentin (1:200, #MA5-11883-A488, Invitrogen). The secondary antibody used was Goat anti-Rabbit Texas Red-X (1:200, #T6391, Invitrogen). For each patient, 10 images were randomly captured at 10× and 20× magnification. Images were captured using a Zeiss Axio Observer Z1 inverted microscope with ZEN 3.6 (blue edition) software.

Cell detection was based on nuclear staining, while relative marker expression was quantified within the cytoplasmic compartment using mean fluorescence intensity in the Alexa Fluor 488 and Alexa Fluor 568 channels. The mean fluorescence intensity corresponds to the average pixel intensity in the respective channel within the segmented cells, as quantified using QuPath Software Version 0.6.0. Data are expressed as the percentage of double-positive EECs (%), averaged per 100 CD31+ EECs.

7. Morphological Observation

Morphological changes in EECs after 48 hours were documented using a 10× inverted phase-contrast microscope.

8. Flow Cytometry

Cells were dissociated into single-cell suspensions and washed with FACS buffer (PBS containing 1% BSA and 0.2 mM EDTA; #15575020, Invitrogen). The suspension was centrifuged at 400 ×g for 5 minutes at room temperature (RT), and the supernatant was completely aspirated. The resulting cell pellet was resuspended in 100 µL of fluorophore-conjugated primary antibodies or IgG isotype controls diluted in FACS buffer and incubated for 20 minutes on ice. Cells were then washed twice with PBS, centrifuged, the supernatant aspirated, and resuspended in 1 mL of FACS buffer (0.5% BSA, 2mM EDTA in PBS 1X).

Surface marker staining included APC-conjugated Fibroblast marker (1:50, #130-123-827, Miltenyi Biotec) and PE-conjugated CD31 (1:100, #303106, BioLegend). For intracellular staining, cells were fixed with

fixation buffer (#ab185917, Abcam) for 10 minutes at RT. Antibodies and isotype controls were diluted in permeabilization buffer (#ab185917, Abcam). Intracellular markers included APC-conjugated NFATC1 (1:100, #sc-7294 AF647, Santa Cruz) and PE-conjugated Vimentin (1:100, #MA1-19656, Invitrogen).

To assess nonspecific binding, the following isotype controls were used: APC Human IgG1 (1:50, #130-113-446, Miltenyi Biotec), PE Mouse IgG1 (1:100, #559320, BD Biosciences), and APC Mouse IgG1 (1:100, #555751, BD Biosciences). Flow cytometry was performed using a BD Accuri C6 Plus cytometer (BD Biosciences), and data were analyzed with FlowJo software version 9 (Tree Star Inc., Ashland, OR).

9. List of Antibodies

**Antibody**	**Vendor**	**Catalog No.**	**Dilution**
**Primary Antibodies**			
4',6-diamidino-2-phenylindole	Invitrogen	#D1306	1:5000
CD144	Invitrogen	#14-1449-82	1:100
Vimentin	Invitrogen	#MA5-11883-A488	1:100
pSMAD2/3	Invitrogen	#PA5-110155	1:50
Slug/Snail	Abcam	#ab85936	1:50
CD31	Abcam	#ab32457	1:200
**Secondary Antibodies**			
Goat anti-Rabbit Alexa Fluor Plus 647	Invitrogen	#A32733	1:200
Goat anti-Mouse Texas Red-X	Invitrogen	#T-862	1:200
Goat anti-Rabbit Texas Red-X	Invitrogen	#T6391	1:200
**Flow Cytometry Antibodies**			
Fibroblast Antibody, anti-human, REAfinity	Miltenyi Biotec	#130-123-827	1:50

PE anti-human CD31 Antibody	BioLegend	#303106	1:100
NFATC1 (7A6)	Santa Cruz	#sc-7294 AF647	1:100
Vimentin Monoclonal Antibody (VI-RE/1), PE	Invitrogen	#MA1-19656	1:100
REA Control Antibody, human IgG1, REAfinity	Miltenyi Biotec	#130-113-446	1:50
PE Mouse IgG1, κ Isotype Control	BD Biosciences	#559320	1:100
APC Mouse IgG1, κ Isotype Control	BD Biosciences	#555751	1:100

10. Quantitative reverse transcription PCR

Total RNA was isolated using the RNeasy Kit (#74106, Qiagen). Quantitative PCR was performed with SYBR Green Master Mix (#A25776, Thermo Fisher Scientific) on a QuantStudio 3 Real-Time PCR System (96-well; #A28567, Thermo Fisher Scientific). As endogenous control gene glyceraldehyde-3-phosphate dehydrogenase (GAPDH) was used. All samples were analyzed in technical triplicate. Ct values were normalized to the housekeeping gene (ΔCt). Target gene expression is presented as relative expression = 1000 × 2^(−ΔCt). Primer sequences are listed below.

**Gene**	**Forward primer (5'-3')**	**Reverse primer (3'-5')**
GAPDH	CATGTTCGTCATGGGTGTGAACCA	ATGGCATGGACTGTGGTCATGAGT
ACTA2	GCCAAGCACTGTCAGGAATC	GGGTACTTCAGGGTCAGGAT
TAGLN	GTCTGGGGAAAGCTCCT	ATGTCTGGGGAAAGCTCCT
CNN1	GAACGTGGGAGTGAAGTACGC	CAGCCCAATGATGTTCCGC
PDGFRB	TCTTTGTGCCAGATCCCACC	AGTGCAACGTCCCCTTTCTT
PECAM	CACCTGGCCCAGGAGTTTC	AGTACACAGCCTTGTTGCCATGT
vWF	GTCGAGCTGCACAGTGACATG	GCACCATAAACGTTGACTTCCA

CD90	GCCTAACGGCCTGCCTAGT	GGGTGAACTGCTGGTATTCTCAT
PDGFRA	TGGCAGTACCCCATGTCTGAA	CCAAGACCGTCACAAAAAGGC

10. Statistical Analysis

All statistical analyses were performed using GraphPad Prism 10 (GraphPad Software, LLC, San Diego, CA, USA) software. Continuous patient-derived variables are presented as median (interquartile range (IQR), 25th-75th percentile), continuous cell culture data as mean with standard deviation (SD), and categorical variables as frequency (%). All patient-derived data were summarized using descriptive statistics. All cell data derived from human samples were tested for normal distribution using normal Q-Q plots and the Shapiro-Wilk test. Normally distributed data were analyzed using an unpaired two-tailed t-test for two-group comparisons and a one-way ANOVA with Bonferroni post hoc correction for multiple groups. All cell culture experiments were performed in biological triplicate. A P-value of < 0.05 was considered statistically significant.
