# Supplementary material for: Fibroelastic Remodelling of the Endocardium on the Right Side of the Heart: Endothelial-to-Mesenchymal Transition in Pulmonary Atresia With Intact Ventricular Septum
Source: Eur J Cardiothorac Surg. 2026 Apr 3;68(4):ezag143. doi: 10.1093/ejcts/ezag143 (PMC13094542; doi:10.1093/ejcts/ezag143)
Supplement: ezag143_Supplementary_Data [file ezag143_supplementary_data.zip › Supplementary_Figures_S1-S5.pdf]

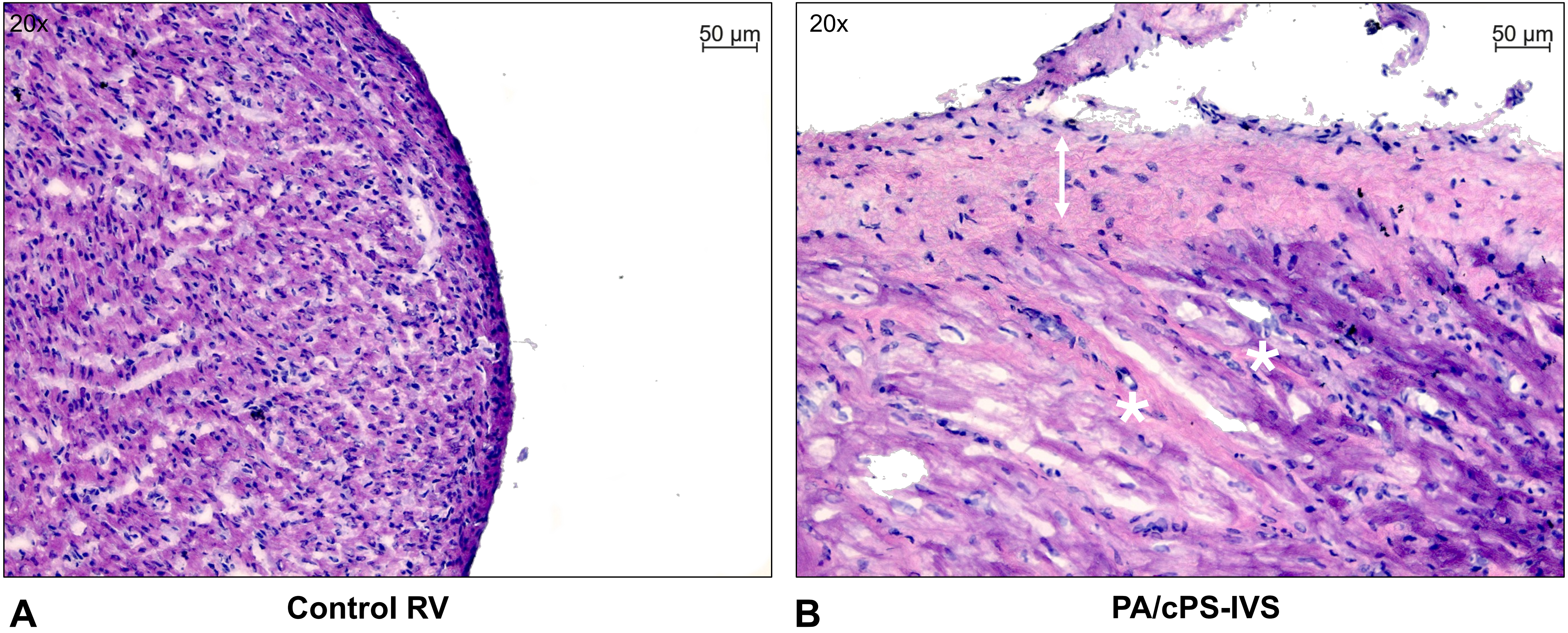

Supplementary Figure S1: Hematoxylin and eosin staining of representative healthy RV control tissue (A) and PA/cPS-IVS sections (B). Nuclei are visualized in blue, and cytoplasm/extracellular matrix in red. In contrast to healthy RV tissue, PA/cPS-IVS tissue exhibits a thickened tissue layer in the subendocardium (arrow). This disproportional layer remains largely avascular and shows low cellular content. There are areas which display an infiltrative growth pattern into the underlying myocardium (stars).

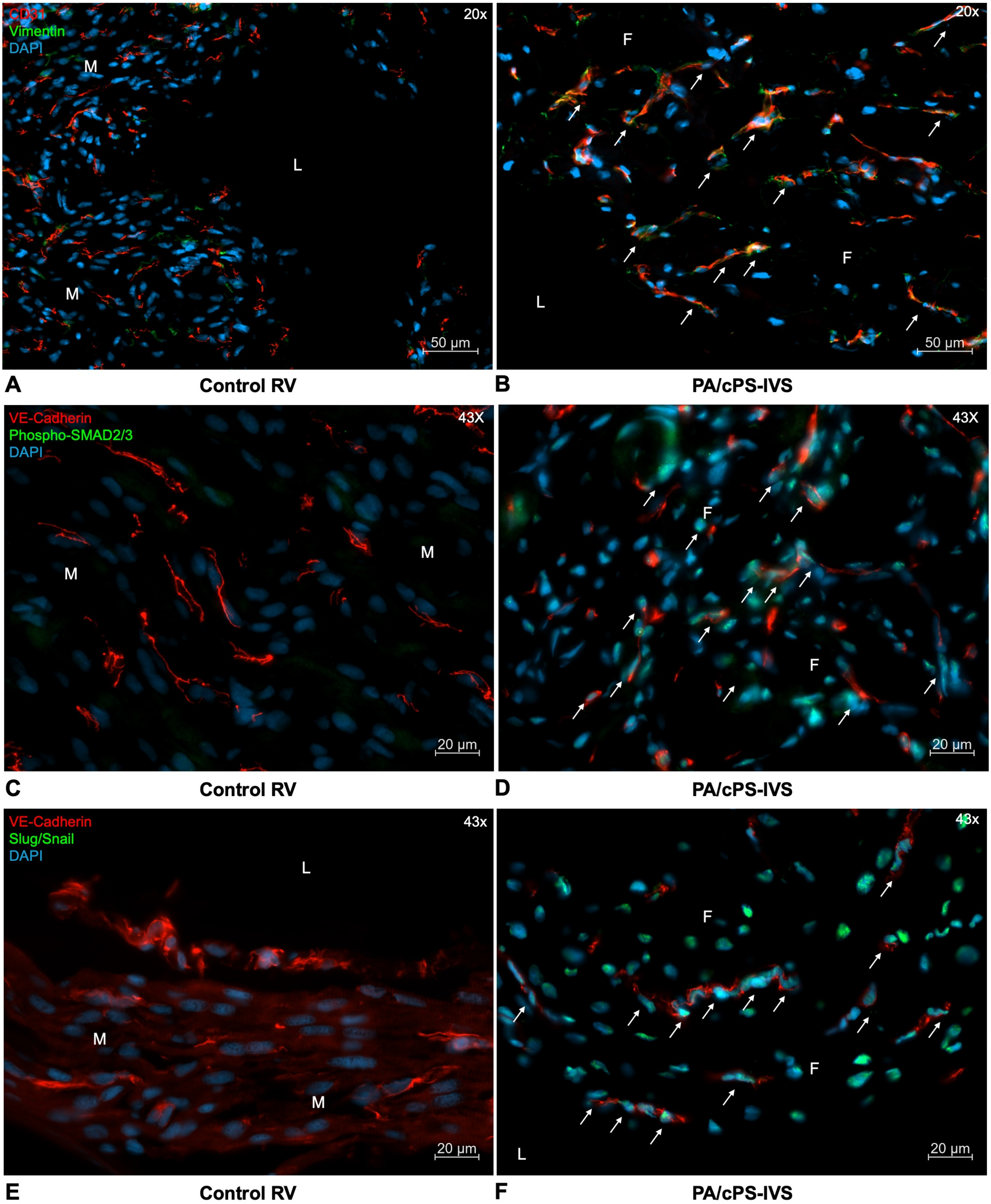

Supplementary Figure S2: Representative immunohistochemical images demonstrate that healthy RV tissue is negative for EndMT. **A-B:** EECs co-expressing CD31 (red) and vimentin (green), appearing yellow when double-positive, indicate EndMT (arrows). Healthy control RV tissue (A) shows no evidence of EndMT, whereas PA/cPS-IVS tissue (B) displays prominent EndMT. **C-D:** In addition, healthy RV tissue (C) shows no nuclear localization of phospho-SMAD2/3 in EECs, in contrast to PA/cPS-IVS tissue (D). EECs marked by VE-cadherin (red) with nuclear phospho-SMAD2/3 expression (green) indicate activation of a TGFβ-mediated signaling pathway (arrows). **E-F:** Furthermore, healthy control RV tissue (E) remains negative, whereas PA/cPS-IVS tissue (F) demonstrates endothelial nuclei co-expressing Slug/Snail, consistent with active subendocardial remodeling via EndMT. EECs expressing VE-cadherin (red) with nuclear localization of the transcription factor Slug/Snail (green) mark active EndMT (arrows). Nuclei are counterstained with DAPI (blue). F = Fibroelastic tissue; L = Lumen; M = Myocardium.

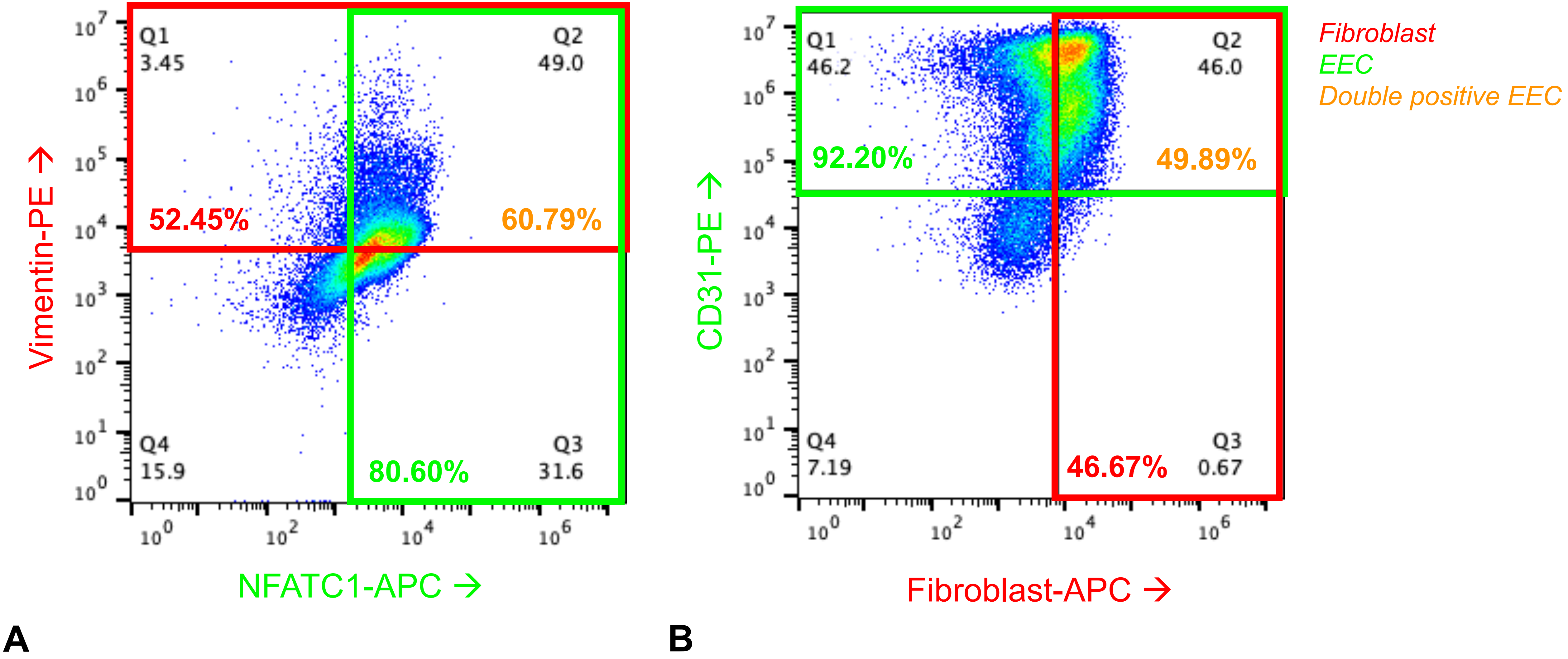

Supplementary Figure S3: Flow cytometry plots of magnetically purified PA/cPS-IVS-EECs demonstrate EndMT at the single-cell level. (A) A substantial proportion of double-positive EECs co-express the endocardial marker NFATC1 and the intracellular mesenchymal marker vimentin. (B) Comparable findings are observed with endothelial CD31 and the fibroblast surface marker. These results highlight that EndMT in the setting of PA/cPS-IVS occurs not only at the endothelial but also at the endocardial-specific level.

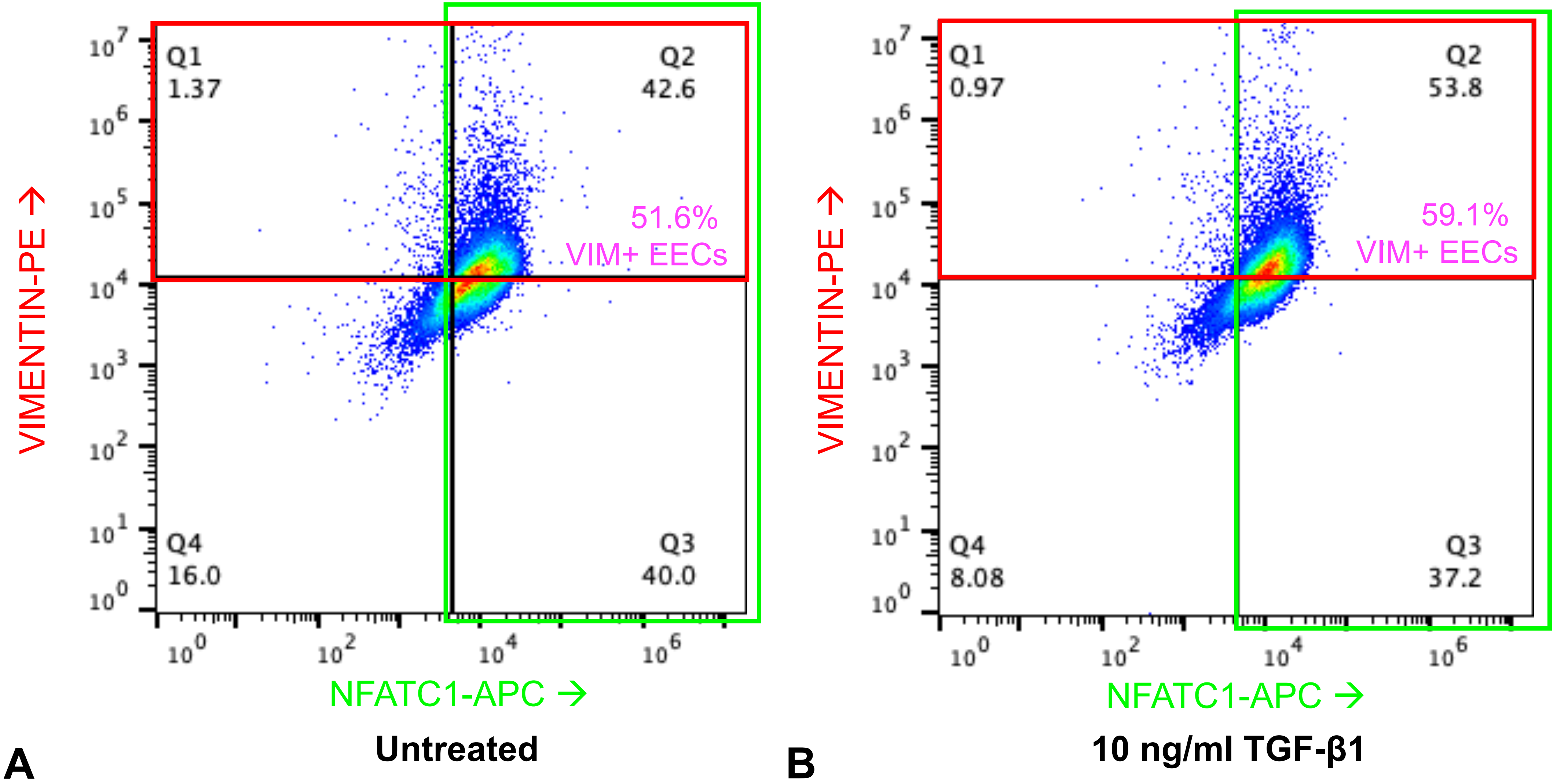

Supplementary Figure S4: Flow cytometry plots of magnetically purified PA/cPS-IVS EECs stained for the mesenchymal marker vimentin and the endocardial-specific marker NFATC1. Following 24-hour exposure to TGFβ1 (10 ng/mL), the proportion of VIM+ EECs (double-positive) increased from 51.6% to 59.1% (fold change 1.15). These results indicate that EndMT in PA/cPS-IVS is, at least in part, mediated by TGFβ-signaling.

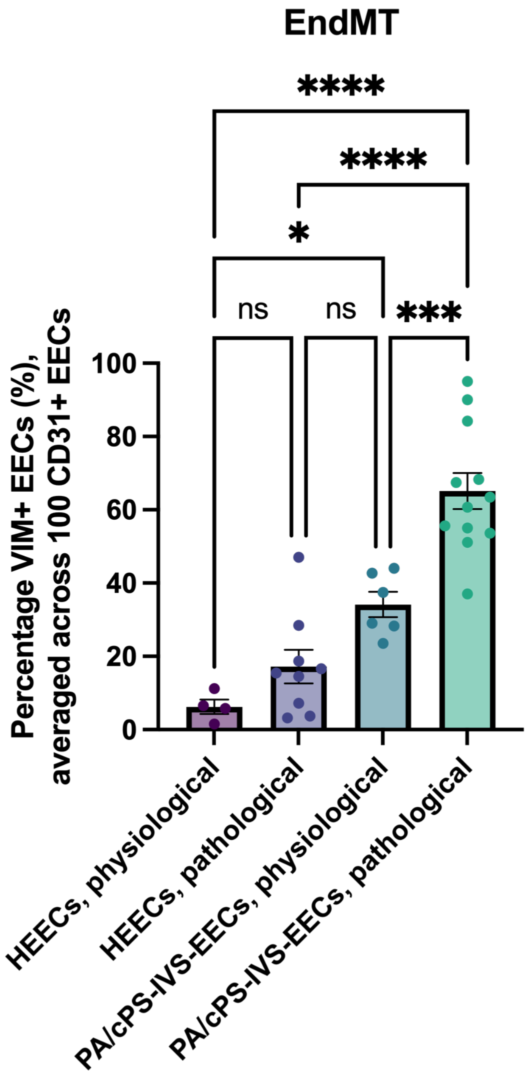

Supplementary Figure S5: Summary graphs show HEECs and PA/cPS-IVS EECs exposed to physiological and pathological flow for 48 hours. PA/cPS-IVS EECs display a unique predisposition compared to HEECs, and distinct vulnerability to EndMT under pathological flow. We observed a significant increase in double-positive EECs in PA/cPS-IVS compared with HEECs under pathological flow conditions. Statistical analysis was performed using ANOVA with Bonferroni post hoc comparisons. Data are expressed as mean ± standard error of the mean. Scatter dot plots correspond to the number (N) of biological experiments performed. Ns $P > 0.05$, $P < 0.05$, ***$P < 0.001$, ****$P < 0.0001$.
